# Bilateral enucleation alters gene expression and intraneocortical connections in the mouse

**DOI:** 10.1186/1749-8104-7-5

**Published:** 2012-01-30

**Authors:** Catherine A Dye, Charles W Abbott, Kelly J Huffman

**Affiliations:** 1 Department of Psychology, University of California Riverside, 900 University Avenue, Riverside, CA 92521, USA; 2 Interdepartmental Neuroscience Program, University of California Riverside, 900 University Avenue, Riverside, CA 92521, USA

**Keywords:** arealization, connectivity, ephrin A5, INCs, neocortex, regionalization

## Abstract

**Background:**

Anatomically and functionally distinct sensory and motor neocortical areas form during mammalian development through a process called arealization. This process is believed to be reliant on both activity-dependent and activity-independent mechanisms. Although both mechanisms are thought to function concurrently during arealization, the nature of their interaction is not understood. To examine the potential interplay of extrinsic activity-dependent mechanisms, such as sensory input, and intrinsic activity-independent mechanisms, including gene expression in mouse neocortical development, we performed bilateral enucleations in newborn mice and conducted anatomical and molecular analyses 10 days later. In this study, by surgically removing the eyes of the newborn mouse, we examined whether early enucleation would impact normal gene expression and the development of basic anatomical features such as intraneocortical connections and cortical area boundaries in the first 10 days of life, before natural eye opening. We examined the acute effects of bilateral enucleation on the lateral geniculate nucleus of the thalamus and the neocortical somatosensory-visual area boundary through detailed analyses of intraneocortical connections and gene expression of six developmentally regulated genes at postnatal day 10.

**Results:**

Our results demonstrate short-term plasticity on postnatal day 10 resulting from the removal of the eyes at birth, with changes in nuclear size and gene expression within the lateral geniculate nucleus as well as a shift in intraneocortical connections and *ephrin A5 *expression at the somatosensory-visual boundary. In this report, we highlight the correlation between positional shifts in *ephrin A5 *expression and improper refinement of intraneocortical connections observed at the somatosensory-visual boundary in enucleates on postnatal day 10.

**Conclusions:**

Bilateral enucleation induces a positional shift of both *ephrin A5 *expression and intraneocortical projections at the somatosensory-visual border in only 10 days. These changes occur prior to natural eye opening, suggesting a possible role of spontaneous retinal activity in area border formation within the neocortex. Through these analyses, we gain a deeper understanding of how extrinsic activity-dependent mechanisms, particularly input from sensory organs, are integrated with intrinsic activity-independent mechanisms to regulate neocortical arealization and plasticity.

## Background

All mammalian behavior is generated and regulated by the nervous system. In humans, the neocortex is the structure within the nervous system that is responsible for the complex integration of information, the ability to utilize language, decision-making, motivation and other high-level emotive-cognitive processes. The complexity of the neocortex emerges during development through arealization, when specific sensory and motor functional units, or areas, are formed and connected to one another and to sub-cortical nuclei through a vast and complex network of intra- and extra-neocortical connections.

Research on the developmental mechanisms that drive arealization has been influenced by two alternative hypotheses. Rakic [1] famously detailed his protomap hypothesis, suggesting that the fates of different neocortical regions were pre-specified in early development, by yet-to-be characterized molecules within the proliferative zone. The alternate model, coined the protocortex hypothesis, emphasized the role of neural activity, via neocortically extrinsic thalamic sensory input, in determining neocortical areal fate [2]. In the past 20 years, a consensus has formed in the field of neocortical developmental biology that both activity-independent cortically intrinsic mechanisms, such as gene expression, and activity-dependent mechanisms that involve input from the sensory organs via the dorsal thalamus interact to form the cortical map. The exact nature of the interaction, however, is not known.

Despite this consensus, most studies focus on one side of the argument or the other. For example, the notion that the developing neocortex is patterned early in development, regardless of driven sensory input, with differential expression of genes during arealization is highly supported [1,3-28]. Specifically, the areal patterning period (APP), or the time in which the major structural features of the developing sensory and motor areas are established, has recently been described and defined as from embryonic day (E) 16.5 to the third postnatal day (P) in mice [28], prior to eye opening and active whisking. Additionally, the absence of thalamocortical afferents (TCAs) during the prenatal portion of the APP (E16.5 to birth), such as in *Gbx2 *or *Mash1 *mutant mice, leaves neocortical gene expression patterns unperturbed, downplaying the role of activity in neocortical patterning [4,5,29]. However, Krubitzer and colleagues demonstrated the impact of the removal of a sensory receptor surface on arealization in an elegant series of very early postnatal enucleation experiments in *Monodelphis domestica*. In these studies, clear expansions of auditory and somatosensory cortical areas into visual cortical regions were described in the adult [30-32]. Moreover, the relative use of different sensory modalities has been correlated with relative areal size [33], indicating that increased activity can also alter the cortical map.

Although changes to the molecular properties of the cortex (via knockout or electroporation studies) and changes in activity from thalamic sensory inputs, including spontaneous lateral geniculate nucleus (LGN) activity [34-36], can independently affect the cortical map either during the APP or during the critical period, it is important to better understand how one factor may impact on the other. Molecular cortical gene expression and spontaneous activity occur together very early in development, during embryogenesis. In the visual system, for example, spontaneous activity from the retina is transmitted to the developing cortex via the thalamus as early as E16 in mice and continues into the postnatal stage [37,38]. Additionally, studies of gene expression patterns suggest that the collaboration between intrinsic and extrinsic factors is extensive; we have previously shown that transcripts for several genes are present throughout embryogenesis and the first three weeks of life [28,39]. It is likely that each may be required in an ongoing fashion to complete successive steps of arealization, including those that are influenced by incoming activity from sensory receptor surfaces. Studies of *chicken ovalbumin upstream promoter transcription factor 1 *(*COUP-TF1*) have demonstrated multiple functions in the development of the somatosensory cortex [40-42]; however, these reports did not address the possible influence of afferent sensory input.

The visual cortex has been used extensively as a model to study aspects of both the protocortex and protomap hypotheses, as changes in visual function can easily be tracked through alterations at multiple levels of the nervous system. Prior to eye opening, during preliminary developmental stages, neocortical gene expression is thought to drive initial arealization and targeting of TCAs and ipsilateral intraneocortical connections (INCs) [6,28,39,43,44], with eye opening and subsequent sensory experience guiding detailed features of visual cortex development [45]. The organization and connections of neurons within the visual cortex of visually impaired animals display sparse abnormal subcortical inputs from the posterior nuclei and the ventral lateral, ventral posterior and anterior thalamic nuclei [46]. Animals lacking visual input display abnormal dendritic spine density as well as altered gene expression and regulation [47,48]. Further evidence demonstrates that bilateral enucleation during early development results in reduced overall brain size, a reduction in the size the of the primary visual cortex yet an increase in the size of the somatosensory cortex, suggesting that the relative sensory activity level determines major features of cortical organization [32,49]. Although bilateral enucleation as an experimental manipulation can affect multiple systems, it has been used extensively as a way to remove retinal input to the developing brain [31,32,48,50].

In the present study we investigated the impact of very early bilateral enucleation on the cortical expression of several genes that have been implicated in arealization or topographic patterning, either through mutation or correlative studies: *Cadherin 8 *(*Cad8*) previously shown to delineate distinct neural pathways and cortical areas [51]; *COUP-TF1*, which is required for proper regionalization and corticospinal motor neuron differentiation [42,43]; *ephrin A5*, strongly expressed in the putative somatosensory cortex and implicated in topographic patterning [28,39,52,53]; *inhibitor of DNA binding 2 *(*Id2*), a well-established gene to study positional identity [54]; *LIM homeobox protein 2 *(*Lhx2*), required for regional specification [55]; and r*etinoic acid receptor related orphan receptor beta *(*RORß, also known as RZRß*), a highly specific marker for the primary sensory areas [7]. We found that, with short-term survival after enucleation from P0 to P10, the dorsal LGN (dLGN), which normally receives direct retinal input, was reduced in size with altered gene expression in the nucleus. Anatomical 'errors' in the postnatal development of INCs were present at the medial somatosensory-visual (S-V) area boundary in the neocortices of enucleated mice. These aberrant INCs stemming from dye placements within the somatosensory cortex co-registered with a positional shift in *ephrin A5 *expression. Our results demonstrate a link between altered sensory input via bilateral enucleation and aberrant gene expression and anatomical development in the neocortex and suggest a possible role of spontaneous retinal activity in the formation of the neocortical S-V area boundary. In this report, we discuss the potential roles that gene expression and sensory activity may play in critical period plasticity as it relates to areal boundaries and physiology.

## Results

Temporary loss of vision early in life profoundly affects the ability to process visual information [56-58]. Although studies in several species have elucidated the physiological consequences of enucleation within the visual cortex, little is known of the effects on the formation and maintenance of INCs between the visual cortex and other cortical sensory regions. Similarly, more information is needed on the relationships between input from sensory receptors and regional and/or laminar-specific expression of genes with known functions in neocortical development. In this report, we explore these issues and present data gained through the use of bilateral enucleation experiments in newborn mice. First, we describe the alteration of gene expression within the thalamus and cortex 10 days after enucleation. *In situ *RNA hybridization (ISH) analyses were used to study the expression of six genes with well-characterized expression during embryogenesis and postnatal periods and known or suspected functions in the topographic organization or arealization of the neocortex: *ephrin A5, Id2, Cad8, COUP-TF1, Lhx2*, and *RZRß *(for review see [28,39]). We then report the alterations of INCs found at the boundary of the visual (occipital) and somatosensory (parietal) cortex of experimental animals and determine the correlation between the observed changes and aberrant *ephrin A5 *expression. We quantified our results through detailed anatomic measurement of INC shifts and density differences in gene expression across different regions of interest (ROIs) in control and enucleated cortical tissue.

### Bilateral enucleation perturbs thalamic gene expression

**Figure 1:**
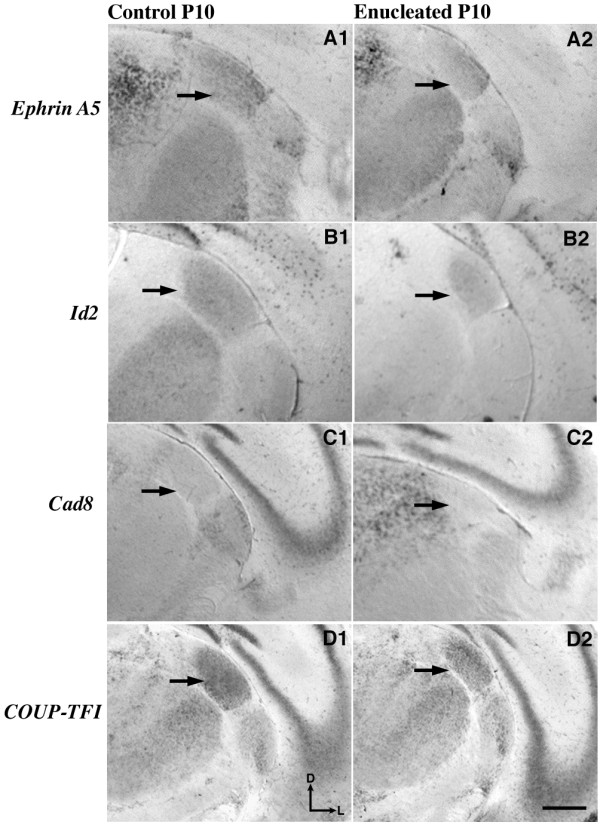
**Bilateral enucleation impacts gene expression in the dLGN**. *In situ *RNA hybridization was used to determine the distribution of transcripts for **(A1-A2) ***ephrin A5*, **(B1-B2) ***Id2*, **(C1-C2) ***Cad8 *and **(D1-D2) ***COUP-TF1 *at P10 in control (A1-D1) and enucleated (A2-D2) mouse brains. All panels are high magnification views of the dLGN and surrounding regions of the dorsal thalamus of one hemisphere after sectioning at 100 μm in the coronal plane. Arrows indicate the dLGN. *Ephrin A5 *is expressed in a gradient in the dLGN at this age that is highest in ventrolateral regions (A1). Enucleation leads to a reduced area of expression of *ephrin A5 *in the nucleus (A2). The expression of *Id2 *is weak but uniform throughout the dLGN (B1), and this expression area is reduced in size by enucleation (B2). *Cad8 *expression in the dLGN was weak at this age in the control (C1) and absent in the enucleated brain (C2). *COUP-TF1 *expression was detected at moderate levels throughout the dLGN (D1) and the region of dLGN expression was reduced following enucleation (D2). Panels A2-D2 demonstrate dLGN shrinkage after enucleation. All sections are oriented with dorsal (D) up and lateral (L) to the right. Scale bar = 500 μm. *Cad8*: *Cadherin-8*; *COUP-TFI*: *chicken ovalbumin upstream promoter transcription factor 1*; dLGN: dorsal lateral geniculate nucleus; *Id2*: *inhibitor of DNA binding 2*; P: postnatal day.

We initiated this study with an examination of the effects of bilateral enucleation on the development of the dLGN of the thalamus (Figure 1; arrows highlight dLGN). Of the four genes we illustrate in the dLGN - *ephrin A5, Id2, Cad8 *and *COUP-TF1 *- only *Cad8 *was not expressed at P10 in the enucleated dLGN (Figure 1C2; arrow). *Ephrin A5 *expression in the dLGN of mice has been documented previously in the first postnatal week [59]; we have extended this finding to include expression in the second postnatal week. At P10, transcripts were found in a gradient of expression that was highest ventrolaterally (Figure 1A1). In enucleated mice, the area of *ephrin A5 *expression was decreased, with expression detected most easily where the gradient was highest (Figure 1A2). *Id2 *message was present at low levels throughout the dLGN with a uniform distribution at P10 (Figure 1B1). In the post-enucleation P10 dLGN, the area of *Id2 *expression was reduced compared to controls, correlating with the overall reduction in size of the dLGN (Figure 1B2). *Cad8 *expression in the dLGN at this age in the control (Figure 1C1) and experimental (Figure 1C2) brains was light, with a comparison of both sections demonstrating the reduction in size of the nucleus. *COUP-TF1 *expression in the dLGN at P10 was observed at strong levels throughout the nucleus (Figure 1D1). To the best of our knowledge, this analysis is the first description of postnatal *COUP-TF1 *expression within this nucleus. In brains from enucleated mice, the area of *COUP-TF1 *expression within the dLGN decreased noticeably in comparison to controls, possibly as a result in the size reduction of the nucleus (Figure 1D2).

Several reports in the literature have documented that eye removal can lead to a loss of volume in the dLGN [60-66]. Our results support those conclusions while demonstrating the rapid, 10-day onset of defects prior to eye opening.

### Neocortical gene expression 10 days after early bilateral enucleation

#### Ephrin A5

**Figure 2:**
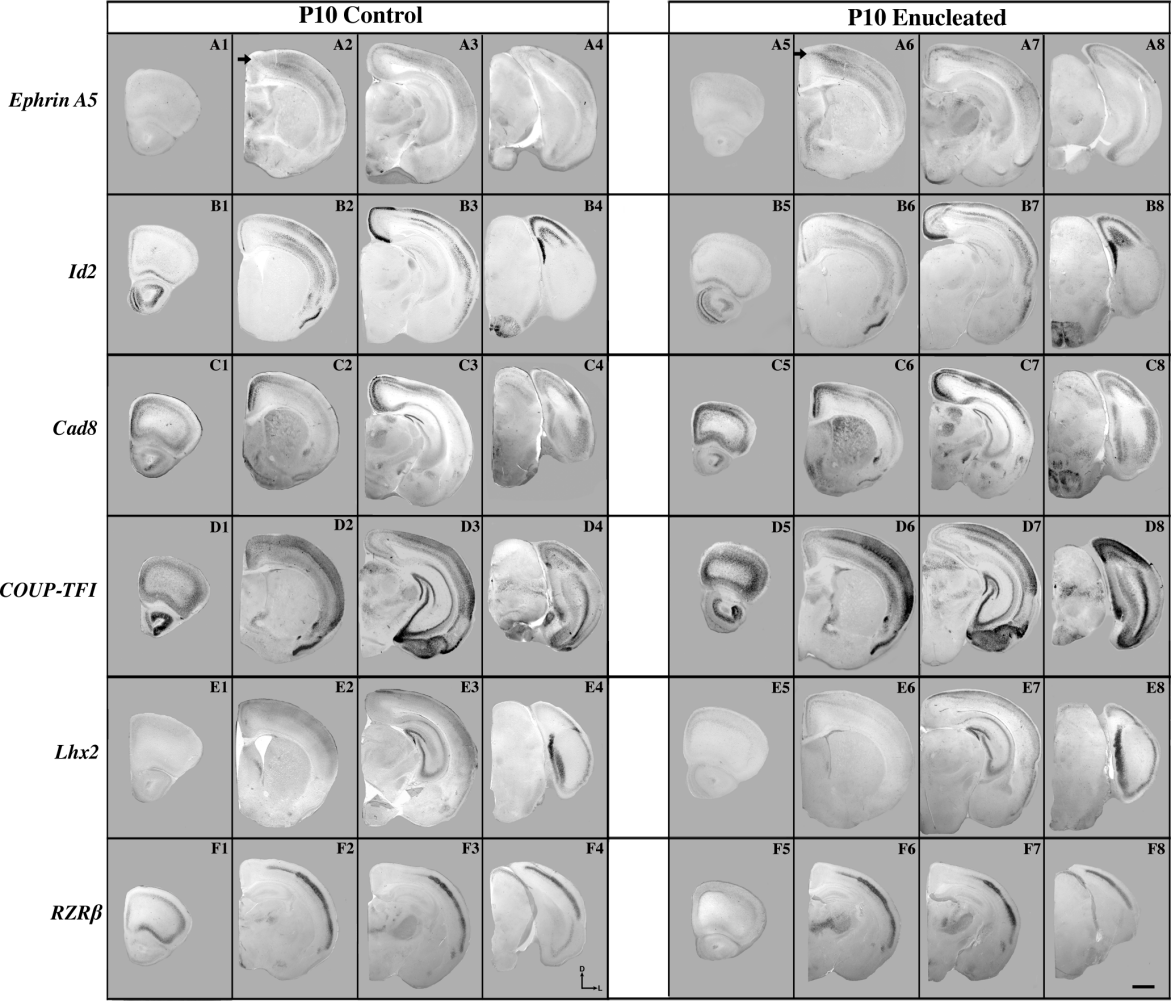
**Neocortical expression of six genes at P10 in the control and enucleated mice**. *In situ *RNA hybridization was used to determine the distribution of transcripts for **(A1-A8) ***ephrin A5*, **(B1-B8) ***Id2*, **(C1-C8) ***Cad8*, **(D1-D8) ***COUP-TF1*, **(E1-E8) ***Lhx2 *and **(F1-F8) ***RZRß *at P10 in control (left panel, columns 1-4) and enucleated (right panel, columns 5-8) brains as labeled. All panels are low magnification views of one hemisphere after sectioning at 100 μm in the coronal plane, shown in a rostral to caudal (left to right) series for each gene. *Ephrin A5 *expression in brains from control animals (A1-A4) was found predominantly in the parietal and occipital cortex, with noted exclusion medially (arrow in A2). In brains from experimental animals, an atypical medial expansion of *ephrin A5 *expression was observed in the parietal cortex (arrow in A6). Control *Id2 *expression was observed throughout the rostral/caudal extent of the cortex with very low levels in layer 4 (B1-B4), with no major changes observed in enucleated brains (B5-B8). *Cad8 *expression was seen in a complex pattern with robust expression in several domains medially and laterally (C1-C4), with no change in expression in enucleated brains (C5-C8). *COUP-TF1 *expression was observed in a gradient throughout the cortex (D1-D4) and the level and distribution of transcripts observed in experimental brains (D5-D8) did not differ significantly from controls. *Lhx2 *is present at low levels in superficial cortical layers in both control (E1-E4) and experimental (E5-E8) brains. *RZRß *showed robust expression in layers 4/5 at all levels of the cortex (F1-F4), and this expression remained unchanged after enucleation (F5-F8). All sections are oriented with dorsal (D) up and lateral (L) to the right. Scale bar = 1,000 μm. *Cad8*: *Cadherin-8*; *COUP-TFI*: *chicken ovalbumin upstream promoter transcription factor 1*; dLGN: dorsal lateral geniculate nucleus; *Id2*: *inhibitor of DNA binding 2*; *Lhx2*: *LIM homeobox protein 2*; P: postnatal day; *RZRß*: *retinoic acid receptor related orphan receptor beta*.

**Figure 3:**
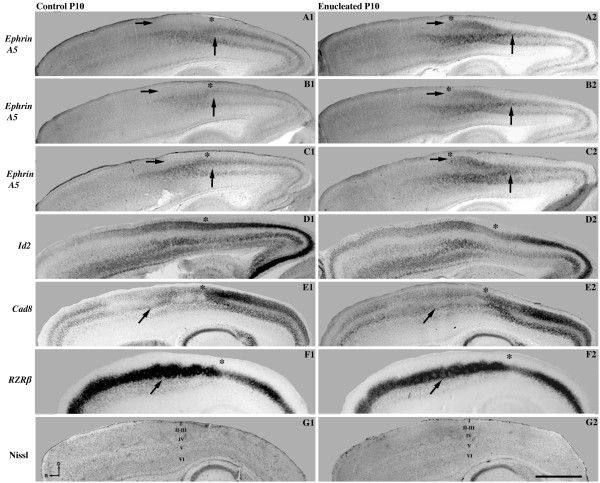
**Early enucleation alters *ephrin A5 *expression in the neocortex**. All panels are high magnification views of 100 μm sagittal sections of P10 brain hemispheres following *in situ *RNA hybridization with probes directed against (**A1-C1**; **A2-C2**) *ephrin A5*, **(D1-D2) ***Id2*, **(E1-E2) ***Cad8 *and **(F1-F2) ***RZRß*. **(G1-G2) **Sagittal sections of 40 μm stained for Nissl substance. Sections shown in A1-C1 and A2-C2 are at a medial location close to the midline, panels D1-D2 are mid-sagittal, and panels E1-E2 and F1-F2 show lateral sections. *Ephrin A5 *expression in the medial parietal cortex of controls was observed in one uninterrupted band of expression within layers 4 and 5 (below left arrows in A1-C1), and no transcripts were detected in the superficial layers (left arrows in A1-C1). In brains from enucleated animals, the expression of *ephrin A5 *was observed in the superficial layers (left arrows in A2-C2) in addition to the band of expression in lower layers (below left arrows in A2-C2). An ectopic expression is also present in caudal layer 4 (right arrows in A2-C2) and not present in the controls (right arrows in A1-C1). Expression patterns of *Id2, Cad8 *and *RZRß *in enucleated brains (D2-F2) show no significant differences from that of controls (D1-F1). Arrows in E1-E2 and F1-F2 denote the location of the barrel field. Cortical layers are labeled by roman numerals in G1-G2. Asterisks indicate the presumptive S-V boundary. All sections are oriented with dorsal (D) up and rostral (R) to the left. Scale bar = 1,000 μm. *Cad8*: *Cadherin-8*; *Id2*: *inhibitor of DNA binding 2*; P: postnatal day; *RZRß*: *retinoic acid receptor related orphan receptor beta*; S-V: somatosensory-visual.

In P10 controls, neocortical *ephrin A5 *expression was largely absent from the frontal/motor cortex (Figure 2A1), but became pronounced in most cortical layers of the somatosensory cortex (Figures 2A2-A3 and 3A1-C1). Transcripts are excluded from medial regions adjacent to the cingulate/retrosplenial areas in control brains (arrow in Figure 2A2); however, within a limited domain of the parietal cortex, *ephrin A5 *expression was observed extending medially towards the cingulate/retrosplenial area in enucleates (arrow in Figure 2A6). Although transcripts were present in enucleated brains in an ectopic position, it does not appear to be an entirely new domain of expression but rather an extension of a more lateral expression domain (compare the region of expression to the right of the arrow in control Figure 2A2 with region of expression extending from the arrow in enucleated Figure 2A6). In the sagittal plane, *ephrin A5 *expression in this region in controls appears as a band of expression within layers 4 and 5 that gradually emerged from the striped pattern present in more caudal areas (Figure 3A1-C1; area between the horizontal and vertical arrows). There was also a noticeable absence of expression in the superficial layers (Figure 3A1-C1; horizontal arrows). In contrast to the controls, *ephrin A5 *expression in enucleated brains displayed robust expression in the upper cortical layers (Figure 3A2-C2; horizontal arrows). The deeper band of expression seen in the control brains remains visible in the enucleated cortex as well (below the horizontal arrows in Figure 3A2-C2). We also observed an ectopic region of *ephrin A5 *expression in caudal layer 4 (Figure 3A2-C2; vertical arrows) where it was absent in controls (Figure 3A1-C1; vertical arrows). Presumptive boundaries between the somatosensory and visual cortex are marked with asterisks in the control and enucleated sections (Figure 3). This boundary in medial sagittal sections processed for *ephrin A5 *expression demonstrates a rostral shift (compare asterisks in Figure 3A1-C1 and 3A2-C2). Sections stained for Nissl substance support laminar distinctions made in the ISH data (Figure 3G1, control; 3G2, enucleated).

#### Id2

Expression of *Id2 *at P10 was observed at very low to absent levels in layer 4 throughout the cortex, but present at varying intensity in layers 2 to 3 and 5 to 6. While frontal/motor regions did not show robust staining, expression was stronger in the more caudal regions in P10 control brains, including the retrosplenial area (Figure 2B1-B4). We did not detect any disturbances in the distribution of *Id2 *transcripts in either the coronal or sagittal plane in P10 enucleated brains (compare Figures 2B1-B4 to 2B5-B8, and 3D1 to 3D2). At this mid-sagittal level, the S-V boundary remains stable after enucleation (compare Figure 3D1 with3D2; asterisks).

#### Cad8

At P10, marked *Cad8 *expression was observed in superficial layers of both the rostral and caudal cortex, overlapping extensively with the visual and motor cortices in controls (Figures 2C1-C4 and 3E1). *Cad8 *expression is also a useful marker to delineate the outlines of the barrels in the somatosensory area (Figure 3E1; arrow). The distribution of *Cad8 *transcripts in experimental brains closely resembled that of controls, including the medial parietal cortex (compare Figure 2C1-C4 with2C5-C8) and the barrel field (compare Figure 3E1 with3E2; arrows). These more lateral sections (Figure 3E1, 3E2, chosen to highlight barrel field position) do not demonstrate the presumptive shift in the areal boundary, as this appears to be, at this age, a more medial phenotype (compare Figure 3E1 with3E2; asterisks).

#### COUP-TFI

Expression of *COUP-TFI *in P10 control brains was observed in a rostrolateral to caudolateral gradient with strong expression in the caudolateral neocortex (Figure 2D1-D4). In brains from enucleated mice, we observed *COUP-TF1 *expression to be comparable to the controls, both in terms of the level and distribution of the message (compare Figure 2D1-D4 and 2D5-D8).

#### Lhx2

At P10, the expression of *Lhx2 *was weak in control brains, but present throughout the cortex, with transcripts found primarily in superficial layers (Figure 2E1-E4). In enucleated animals, cortical expression of *Lhx2 *showed no major alterations in either the distribution or accumulation of transcripts (Figure 2E5-E8).

#### RZRß

In control brains, *RZRß *is strongly expressed in layer 4 of the visual, auditory and somatosensory areas at P10 (Figures 2F1-F4 and 3F1) and can be considered a marker for the primary areas [39]. We did not detect changes in neocortical expression between controls and enucleated mice (compare Figure 2F1-F4 to 2F5-F8 and Figure 3F1 to 3F2). These more lateral sections (Figure 3F1, 3F2, chosen to highlight barrel field position) do not demonstrate the presumptive shift in areal boundary, as this appears to be, at this age, a more medial phenotype (compare Figure 3F1, 3F2; asterisks).

### Early bilateral enucleation alters intraneocortical connectivity at the S-V border

**Figure 4:**
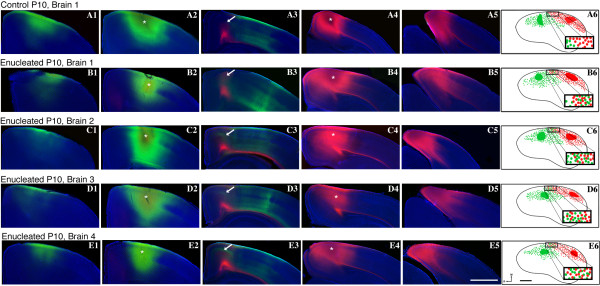
**Ectopic INCs ten days after early enucleation**. **(A1-A5**, **B1-B5**, **C1-C5**, **D1-D5**, **E1-E5) **Rostral to caudal series of 100 μm coronal sections of P10 hemispheres following placement of DiA or DiI crystals in putative visual or somatosensory cortices to label INCs and **(A6-E6) **flattened reconstructions of DPLs and INCs in control and enucleated brains. In the representative control brain, retrogradely labeled cells from a DPL in the parietal somatosensory cortex are found rostral and caudal to the DPL (green labeling in A1-A3) and do not overlap with cells or axons labeled by an occipital visual cortex DPL (red labeling in A3). The arrow in A3 indicates the region of non-overlap between the labeling fields of the two areas, which represents the medial S-V areal boundary at P10. In brains from enucleated mice, retrogradely labeled cells from the somatosensory DPL are found rostral and caudal to the DPL (green labeling in B1-B3, C1-C3, D1-D3, E1-E3). Labeled cells are also found in more medial positions not seen in controls, resulting in an overlap with cells labeled by a visual DPL (regions at and below arrows in B3, C3, D3, E3). This extension of the green label in B3, C3, D3, E3 (arrows) may represent a shift of the medial S-V boundary. These retrogradely labeled green cells project to the somatosensory DPL, and are present in a region normally devoted to visual cortex. This shift of the medial boundary of somatosensory cortex is illustrated in the lateral view reconstructions in B6, C6, D6, E6 (compare boxed regions and their magnified insets in B6, C6, D6, E6 with boxed region and inset in A6 where no overlap exists). Colored regions in reconstructions: green: somatosensory DPL; red: visual DPL; small green dots: retrogradely labeled cells from parietal somatosensory DPL; small red dots: retrogradely labeled cells from occipital visual DPL; rectangular boxes: highlight S-V border region, magnified in inset. In rostral (left) to caudal (right) serial sections, dorsal is up and medial is to the left. In the reconstructions, medial (M) is up and rostral (R) is to the left. Scale bars = 1,000 μm. Asterisks indicates DPL. DiA: 4-(4-(dihexadecylamino)styryl)-N-methylpyridinium iodide; DiI: 1,1'-dioctadecyl-3,3,3',3'-tetramethylindocarbocyanine perchlorate; DPL: dye placement location; INC: ipsilateral intraneocortical connection; P: postnatal day; s: somatosensory areas; S-V: somatosensory-visual; v: visual cortex.

**Figure 5:**
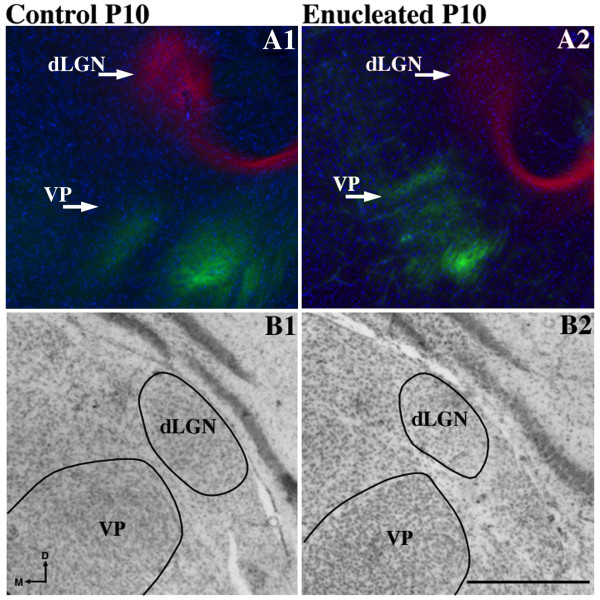
**P0 enucleation does not dramatically alter the sensory thalamic afferent position at P10**. High magnification views of 100 μm coronal hemisections of DAPI stained DiI/DIA labeled sections and Nissl sections through the thalamus of **(A1-B1) **P10 control and **(A2-B2) **enucleated brains. Retrograde thalamocortical labeling in the dLGN and VPN was observed for both (A1) control and (A2) experimental brains, which confirmed that DPLs were within the visual and somatosensory cortices and demonstrated no effect of enucleation. Alternate sections from P10 (B1) control and (B2) enucleated brains stained for Nissl substance to aid in the verification of the nuclear retrograde label. The dLGN and VPN are indicated with text and outlined in the Nissl sections. Dorsal (D) is up and medial (M) is to the left. Scale bar = 500 μm. DAPI: 4',6-diamidine-2-phenylindole dihydrochloride; dLGN: dorsal lateral geniculate nucleus; DiA: 4-(4-(dihexadecylamino)styryl)-N-methylpyridinium iodide; DiI: 1,1'-dioctadecyl-3,3,3',3'-tetramethylindocarbocyanine perchlorate; DPL: dye placement location; P: postnatal day; VPN: ventral posterior nucleus.

Cortical areas and their boundaries are measured and defined using several criteria. In addition to architectonic description using patterns of Nissl or cytochrome oxidase staining, the arrangement of INCs within and among sensory areas has been used to mark areal boundaries in the cortex [6,28,39]. In order to determine the effects of early bilateral enucleation on the development of INCs, 1,1'-dioctadecyl-3,3,3',3'-tetramethylindocarbocyanine perchlorate (DiI) and 4-(4-(dihexadecylamino)styryl)-N-methylpyridinium iodide (DiA) were utilized in postmortem brains of P10 control mice and mice enucleated at birth. INC tracing from a DiA dye placement location, or DPL, in the parietal somatosensory cortex revealed retrogradely labeled cells observed in positions rostral and caudal to the DPL in control mouse brains (Figure 4A1-A5; green, DPL starred in A2). When DiI was placed in occipital visual cortex of controls, INCs were found at rostral and caudal locations relative to the DPL (Figure 4A3-A5; red, DPL starred in A4) with some uptake in axons in A3. In controls, a mixing or overlap of cells at the S-V boundary was never observed (Figure 4A3; arrow and reconstructed in A6, box with inset). In P10 brains of mice enucleated at P0, the global organization of INCs resembled that of the controls (Figure 4B1-B5, 4C1-C5, D1-D5, E1-E5), with the exception of a distinct mixing of labeled neurons from the somatosensory and visual DPLs, which created a nondescript, overlapping medial S-V boundary region demonstrated both in the raw data (Figure 4B3-E3; arrows) and the cortical reconstruction (Figure 4B6-E6; boxes with insets). This mixing is produced from an atypical extension of labeling from the somatosensory DPL into the rostral medial portion of the visual cortex. Ten days without eyes does not demonstrate global changes in the sensory map (compare Figure 4A6, 4B6-E6); however, the positions of INCs at the medial S-V boundary are impacted in this acute enucleated period prior to eye opening. Despite the shift of INCs at the S-V boundary in the enucleated mouse neocortex, the TCAs appear grossly unaltered (compare Figure 5A1 and 5A2; arrows designate retrogradely labeled cells in the LGN and ventral posterior nucleus (VPN) in both representative cases, verified by Nissl stain in B1-B2). Additionally, tracing of TCAs demonstrated consistent and accurate dye placement, ensuring that the altered areal boundaries observed in the enucleated subjects were not artifacts of erroneous dye placements.

### Co-registration of INCs and gene expression after bilateral enucleation

**Figure 6:**
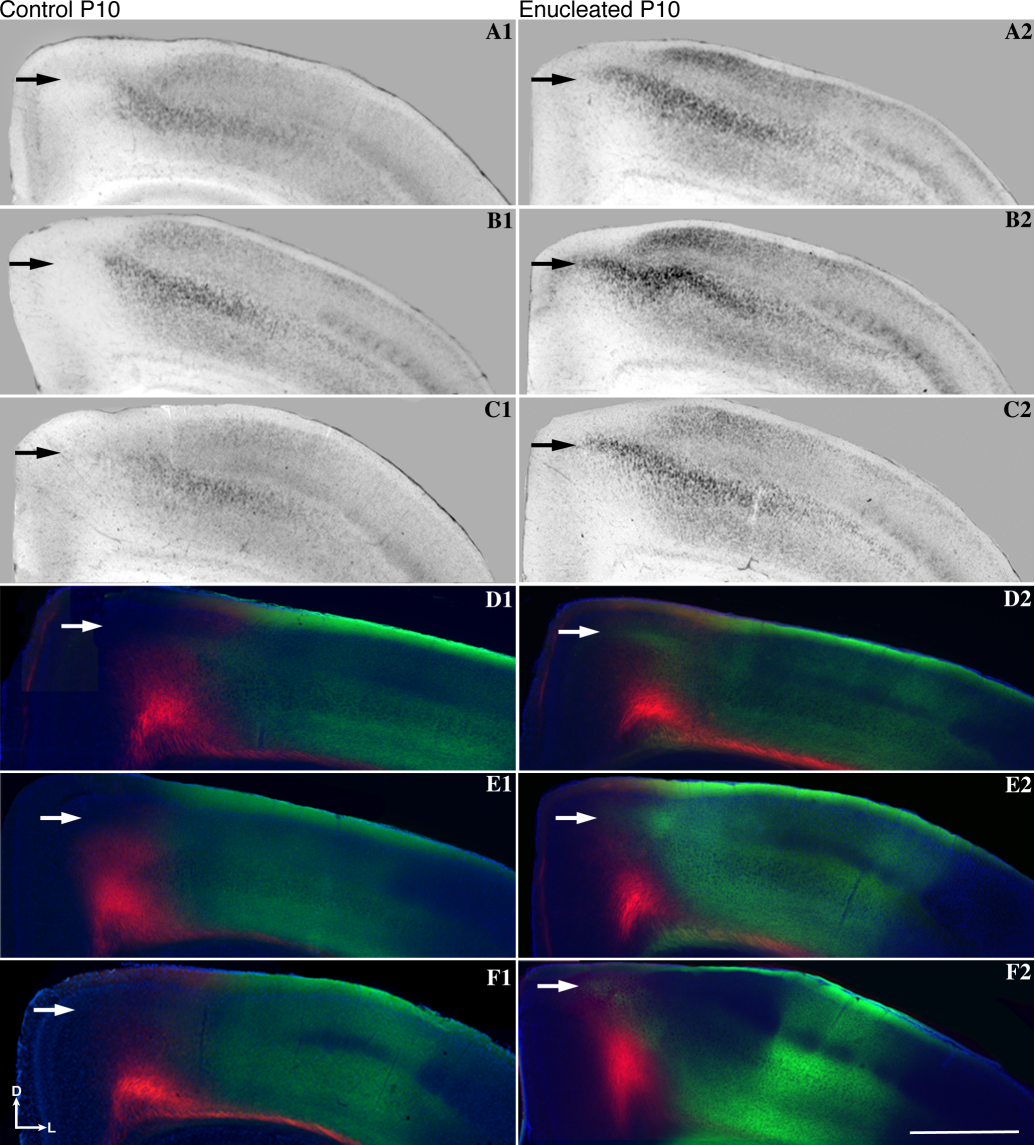
**Co-registration of gene expression and INCs at the S-V boundary in P10 control and enucleated mice**. High magnification views of 100 μm coronal hemisections following *in situ *RNA hybridization with a probe directed against *ephrin A5 *in P10 **(A1-C1) **control and **(A2-C2) **enucleated mice. Tracing of INCs after placement of DiA or DiI in putative visual and somatosensory cortex in P10 **(D1-F1) **control brains and **(D2-F2) **brains from enucleated mice. Sections in A1-A2, B1-B2, C1-C2 were matched with D1-D2, E1-E2, F1-F2 sections using anatomical landmarks. In control brains, *ephrin A5 *expression is excluded from medial regions of the parietal cortex (arrows in A1-C1). This region does not possess any retrogradely labeled cells from dye placement in the somatosensory cortex (absence of green labeling at arrow in D1, E1, F1) but does contain retrogradely labeled cells and axons from a visual DPL (red labeling at and below arrow in D1, E1, F1). Following enucleation, both *ephrin A5 *expression (A2-C2) and cells projecting to the somatosensory cortex (green labeling at arrow in D2-F2) were observed medially in the parietal cortex, indicating that gene expression and INCs are co-registered. Dorsal (D) is up and lateral (L) is to the right. Asterisks indicate the medial limit of *ephrin A5 *expression (A1-C1, A2-C2) or labeling from a somatosensory DPL (D1-F1, D2-F2). Scale bars = 1,000 μm. DiA: 4-(4-(dihexadecylamino)styryl)-N-methylpyridinium iodide; DiI: 1,1'-dioctadecyl-3,3,3',3'-tetramethylindocarbocyanine perchlorate; DPL: dye placement location; INC: ipsilateral intraneocortical connection; P: postnatal day; S-V: somatosensory-visual.

By removing both eyes at birth, we not only altered the expression of at least one well-characterized guidance molecule, *ephrin A5*, but also disturbed the arrangement of INCs at the S-V boundary. In order to better understand the relationship between these two phenotypes, we co-registered *ephrin A5 *gene expression with the aberrant INCs at the S-V boundary (Figure 6). Expression of *ephrin A5 *appeared in the parietal cortex of control brains with pronounced medial exclusion in the superficial layers, whereas the same region in the enucleated mice displayed a medial expansion of the *ephrin A5 *expression pattern (compare Figure 6A1-C1 and 6A2-C2; arrows). The S-V boundary visualized with DiI and DiA tracing of INCs in the controls revealed an extension of somatosensory labeling in the enucleates (Figure 6D2, 6E2, F2; green labeling, from three DiA DPLs in the somatosensory cortex), revealing an overlapping area of mixed red and green retrogradely labeled cells that was not present in the controls (compare Figure 6D1-F1 and 6D2-F2; arrows). This region of altered INC patterning (Figure 6D2-F2; arrows) co-registered with the expansion of *ephrin A5 *expression observed in enucleated brains (Figure 6A2-C2; arrows). In neocortices of enucleated mice, somatosensory cortex INCs are shifted medially into a territory that would normally be visual cortex, and this shift is observed in both gene expression and INC data (Figure 6, compare control A1-F1 and enucleated A2-F2; arrows).

**Figure 7:**
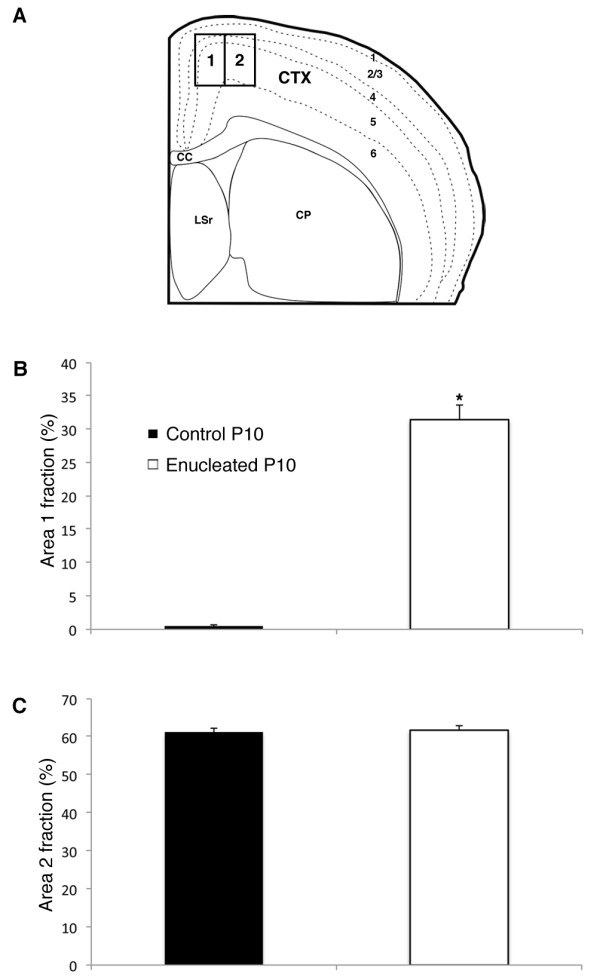
**Analysis of *ephrin A5 *expression in two ROIs in the neocortex of control and enucleated mice**. **(A) **Medial parietal cortex regions of interest overlaid on a representative coronal brain illustration. **(B) **Percent area fraction of cells expressing *ephrin A5 *within ROI1, located within the medial parietal cortex. A sharply increased percentage of transcript is observed in enucleated cases (31.46 ± 2.2%; df = 12; n = 7) when compared to control animals (0.49 ± 0.18%; df = 12; n = 7; *P *< 0.001; two-sample t-test). **(C) **Percent area fraction of cells expressing *ephrin A5 *within ROI2, located immediately lateral to ROI1. Percentage of *ephrin A5 *transcripts within the putative somatosensory cortex of enucleated animals (61.77 ± 1.18%; df = 12; n = 7) are not significantly different from controls (61.29 ± 1.00%; df = 12; n = 7; *P *= 0.47). CC: corpus callosum; CP: caudoputamen; CTX: cortex; df: degrees of freedom; LSr: lateral septal nucleus; ROI: region of interest.

In order to quantify our results, we measured the density of cortical gene expression in two ROIs in control and P10 enucleated brain tissue using the software program ImageJ. For each case analyzed, one ISH section was chosen at a specific rostral-caudal level, and this anatomical level was matched across all cases. The border between ROI 1 and 2 was set at the medial boundary of *ephrin A5 *expression, spanning across layers 4 and 5 in coronal sections at the rostral-caudal level shown in Figure 6. Each ROI was 750 × 600 μm, with the locations drawn in Figure 7A. Two sample t-test analyses were conducted to assess group differences in ISH expression density, expressed as per cent area fraction, between ROI 1 and 2 in both control and enucleated tissue. A statistical comparison of transcript density showed a significant increase in ROI 1 of enucleated cases (31.46 ± 2.19%; n = 7) when compared to control tissue (0.49 ± 0.18%; n = 7; *P *< 0.001; Figure 7B). The same comparison made between ROI 2 of enucleates (61.29 ± 1.00%; n = 7) and controls (61.77 ± 1.18%; n = 7; *P *= 0.47; Figure 7C) revealed no significant differences. ROI 1 corresponds to the region of aberrant *ephrin A5 *expression highlighted in Figure 6.

**Figure 8:**
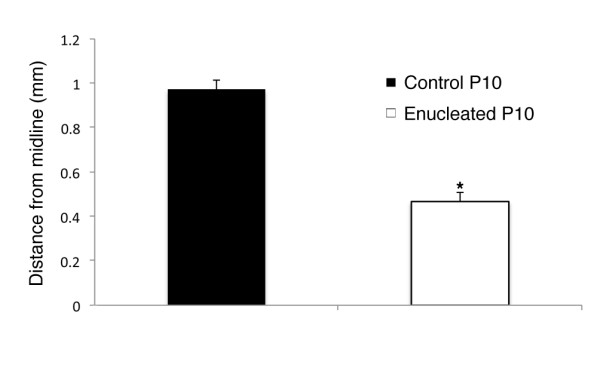
**Analysis of somatosensory INC position within the medial parietal cortex of control and enucleated mice**. Following placement of DiA or DiI crystals into the somatosensory cortex and time for retrograde transport of tracer, the straight-line distance from the midline edge of layer 1 of the neocortex to the most medial retrogradely labeled cell was measured using precision micrometry. Enucleated animals exhibited a distinct extension of labeling from the somatosensory cortex into the visual cortex, resulting in a reduced distance to midline (0.46 ± 0.04 mm; df = 12; n = 7) when compared to controls (0.97 ± 0.04 mm; df = 12; n = 7; *P *< 0.01; two-sample t-test). DiA: 4-(4-(dihexadecylamino)styryl)-N-methylpyridinium iodide; DiI: 1,1'-dioctadecyl-3,3,3',3'-tetramethylindocarbocyanine perchlorate; INC: ipsilateral intraneocortical connection.

To quantify the observation of a shift in somatosensory INCs at the S-V boundary, we measured the position of the most medial labeled cell resulting from a somatosensory dye placement as the distance from the cortical midline edge using a high-precision micrometer within the Zeiss Axiovision software program (Carl Zeiss Inc., North America). All cases included in these analyses had somatosensory DPLs that were precisely and reliably placed using a dye placement grid [6]. Enucleated animals exhibited a distinct extension of labeling from the somatosensory cortex into what would normally be the visual cortex, resulting in a reduced distance to midline (0.46 ± 0.04 mm; n = 7) when compared to controls (0.97 ± 0.04 mm; n = 7; *P *< 0.01; Figure 8).

Collectively, our data indicate that alterations in gene expression and INC patterns caused by a complete loss of vision from enucleation are inter-related phenomena. Loss of vision early in life resulted in atypical expression of *ephrin A5 *in a specific domain of the parietal cortex, a location that correlates with the natural S-V cortical boundary. Changes in incoming sensory information, as with removal of the eyes, can produce selective alterations in cortical gene expression that may have relevance for the development and refinement of neural circuits at developing areal boundaries.

## Discussion

The mechanisms underlying neocortical arealization have become a central focus for many researchers in the field of developmental neurobiology. The protocortex versus protomap debate has been extensive, with the existence of data supporting both arguments. Despite our recent extensive lifespan analysis of INC development in the mouse [28,39], how activity or genetic mechanisms alter the development of murine INCs is an understudied area of research. Of particular interest to those in the fields of neocortical development, arealization or plasticity is how spontaneous neural activity may modify and interact with genetically mediated patterning and arealization in the neocortex. With this report, we attempt to better understand ways in which activity-dependent and activity-independent processes interact at the end of and after the APP in the mouse neocortex. We eliminated all retinal input to the thalamus and neocortex at birth via bilateral enucleation, and examined both genetic (gene expression) and anatomical (INC, Nissl) markers of arealization 10 days later. We hypothesized that bilateral enucleation prior to mouse eye opening would impact inter-areal boundary development and intrinsic gene expression in the neocortex. Our data support these hypotheses and, interestingly, demonstrate acute enucleation-induced changes in both gene expression and INCs only at the natural boundary between the somatosensory and visual cortex. Specifically, data gathered from *in situ *RNA hybridization of six genes revealed a distinct shift in *ephrin A5 *expression in enucleated mice at the S-V boundary. Complementary data following DiI or DiA crystal placement within the visual and somatosensory cortices of enucleated mice revealed that INC formation at the medial S-V areal boundary exhibited an atypical mixing of retrogradely labeled cells at the boundary, with cells labeled from somatosensory dye placements extending into an abnormal location. This extension of somatic labeling correlated with an extension of *ephrin A5 *expression in the enucleated mouse neocortex. These changes occurred without major alteration in the thalamic projections from the LGN and the VPN. Our data suggest that early input from intact retinae may be required for precise gene expression at the boundaries between sensory areas. Although bilateral enucleation as a technique may have additional effects on the developing nervous system, such as altered activity within the LGN or the thalamocortical loop [35], our observed effects in the cortex represent novel insights into potential interactions of intrinsic molecular processes and sensory input. We believe that the interaction of input and gene expression is important for the generation and maintenance of areal boundaries, furthering ideas from previous reports which have proposed that interplay between the two stimulate activity-dependent circuit maturation [8,45,48,67-69]. We have shown that a shift in intraneocortical connections can occur as early as 10 days after a day-of-birth bilateral enucleation, prior to natural eye opening when the majority of driven activity begins. This shift in developing somatosensory INCs was correlated with changes in gene expression. Here we suggest possible roles for early sensory input and neocortical gene expression in arealization and the regulation of critical periods that limit plasticity.

### Effects of short-term enucleation on the dorsal thalamus and TCAs

We have demonstrated that neonatal eye removal at P0 leads to a loss of volume in the dLGN in just 10 days (Figure 1) without any significant shift in thalamic afferents to either the occipital (visual) or parietal (somatosensory) cortex (Figure 5). Although our results confirm previous observations of post-enucleation dLGN shrinkage [60-66], the dLGN was still present and thalamic afferents were still intact. The viability of these connections and the unaltered pattern in enucleated brains is most likely due to the short post-enucleation time period and because the day-of-birth enucleations occurred after the thalamic afferents made contact with the cortical plate [70]. Concomitant with volume loss, dLGN phenotypes in mice, ferrets and cats include a loss of neurons and glia, decreased soma size, delayed dendritic pruning, altered synaptic morphology and absent laminarization [60,62,63,71-73]. We have significantly extended these findings to include alterations in gene expression levels of *ephrin A5, Id2, Cad8 *and *COUP-TFI *(Figure 1) in the enucleated mouse dLGN. These genes have previously been shown to control aspects of differentiation that are abnormal in the thalamus of enucleated animals. *Ephrin A5 *signaling, for example, has been shown to control hippocampal neurogenesis and synaptogenesis [74,75] in addition to the regulation of corticothalamic topography [66,76]. Loss of *COUP-TF1 *and *Id2 *disrupts neuronal differentiation and inhibits axon outgrowth, respectively [41,42,77]. Finally, studies of *Cad8 *mutants reveal altered synaptic function within dorsal root ganglia [78]. Thus, the observed thalamic phenotypes in different mammalian species following enucleation could result, in part, from changes in the expression of these genes.

### Effects of short-term enucleation on neocortical gene expression

The neocortex of enucleated mice had an altered domain of *ephrin A5 *expression in the medial parietal cortex at P10 (Figures 2 and 3). Although the nature of the relationship between spontaneous retinal activity and *ephrin A5 *expression in the parietal cortex remains unknown, the loss of the eyes may initiate abnormal cortical activity leading to abnormal expression of *ephrin A5 *at this age. This abnormal expression of a gene (*ephrin A5*) related to the development of somatosensory topography in the literature [53] may play a role in the post-enucleation expansion of the somatosensory cortex into visual cortical regions, as described in other studies [30-32]. A large-scale change in the developing visual system accomplished through bilateral enucleation could induce this molecular change at the S-V boundary, later facilitating the physiological expansion of the somatic cortex in the occipital lobe. Alternatively, we may be observing a delay of *ephrin A5 *expression triggered by early enucleation, resulting in a more immature S-V boundary at P10. This idea is consistent with other reports suggesting that early visual deprivation can extend the critical period for ocular dominance plasticity [79-83] and other features of neocortical development. Additionally, it is possible that disturbances in local visual cortical circuits, arising from a lack of retinal activity, may affect transcription at the S-V boundary, as retinal waves have recently been demonstrated to modulate activity in the early postnatal neocortex [84,85]. Another potential explanation is that abnormal patterning of ephrin A5 and ectopic INCs at the natural S-V boundary could be induced by abnormal activity arising from cholinergic brainstem afferents, or within the LGN or the thalamocortical loop as neural activity of these structures may be involved in cortical patterning independent of retinal input [35]. However, our data complement that of Majdan and Shatz [47], who reported alterations in gene regulation resulting from a decrease in visual activity via dark rearing. They demonstrated that activity-dependent circuit maturation and proper critical period development depended on both experience and genetic factors. *Ephrin A5 *expression observed in our P10 enucleates may represent one mechanism underlying the extended critical period observed in other model systems.

An additional possible explanation for our phenotype at the S-V boundary includes a cell death-induced reduction in layer 4 of the caudal visual cortex. This idea suggests that the somatosensory cortex remains stable in size, but appears to expand next to a shrinking caudal cortex. However, we do not subscribe to this explanation, as it is not consistent with our data, where a normal layer 4 remains present in the P10 visual cortex. In summary, the observed change in the S-V boundary could represent an expansion of that region into occipital cortex, a delay in boundary maturation or appear as a caudomedial shift due a reduction in the size of the caudal cortex. Another possible mechanism to explain the post-enucleation boundary shift lies in neurophysiology, where the release of sub-threshold activation from inhibition could occur after deafferentation, resulting in neuronal unmasking and areal expansion across natural boundaries. This potential physiological mechanism may or may not be mediated genetically [86].

### INCs in the enucleated mouse neocortex

In the wild-type P10 mouse, somatosensory and visual cortex INC development is well defined, with a distinct segregation evident at areal boundaries (Figure 4A3, 4A6). Spontaneous retinal wave activity and trans-eyelid derived sensory information have been shown to play a role in early developmental processes [66,87-89], and enucleated animals lacking both exhibited reduced refinement medially along the S-V border (Figure 4B6-E6). Hence, day-of-birth bilateral enucleation resulted in altered patterning at areal boundaries, suggesting that early sensory input from the eyes and thalamus may be responsible for cortical boundary refinement and maintenance, even in the first 10 days of life prior to natural eye opening. This early anatomical change at the boundary, coupled with the observed change in *ephrin A5 *expression, may be an underlying substrate for cross-modal shifts observed in other animal models deprived of early visual input. Although our data demonstrate a shift in INCs at the S-V boundary, we cannot speak to the functional nature of the boundary in our enucleated mice. Electrophysiological studies would be needed to determine whether the ectopic somatosensory INCs actually reflect a change in the functional boundary.

### Co-registration of gene expression and INCs following loss of peripheral activity

If the protocortex and protomap hypotheses are both valid, then how does neural activity from the sensory receptors interact with and impact intrinsic genetic mechanisms? Do the mechanisms underlying the models represent orthogonal, non-correlated processes? We believe that gene expression is involved in axon guidance of INCs during development and arealization [6,28,39]. By examining the effects of a large-scale perturbation of sensory system development on the expression of developmentally significant genes and areal boundary development as visualized through INCs, we have begun to characterize the relationship between total vision loss, *ephrin A5 *expression and areal boundary refinement.

Neonatal bilateral enucleation prompts *ephrin A5 *transcripts in the P10 neocortex to adopt a pattern not normally seen at this age. We suspect that the shift in expression triggers the remodeling of neuronal connectivity and results in a poorly defined areal border, and subsequent shifts in cortical physiology. *Ephrin A5 *has long been known to mediate several aspects of axon outgrowth and pathfinding, and the expression of its receptor has been shown to be activity-responsive [9,90-93]. The co-registration of dye-visualized INCs and expression of the key developmental protein ephrin A5 at the medial areal boundary in the enucleated mouse model indicates a possible role for sensory input as a shaping force that refines cortical boundaries initiated earlier by gene expression. However, the ectopic expression of *ephrin A5 *expression at the medial S-V boundary at P10 may not be related to the aberrant INCs at this age, despite a previously demonstrated link between gene expression and INC development shown in normal and mutant mice [6,28,39]. It is possible that growth factors that induce INC targeting may function independently from the expression of *ephrin A5*. Further studies need to be conducted, particularly in *ephrin A5 *mutant mice, to better elucidate the role of this gene in the formation and maintenance of the S-V boundary during the APP and critical period.

## Conclusions

Removing both eyes very early in postnatal development alters gene expression and axonal projections at sensory area boundaries. We hypothesize that neocortical gene expression may be involved in the maintenance of normal areal boundaries in early postnatal life during the critical period, after the major aspects of patterning are complete. The observation that intensity of neocortical gene expression, particularly *ephrin A5*, decreases with age [39], declining alongside closures of sensory critical periods, suggests that gene expression may play a role in the regulation of critical period timing and cortical plasticity. This documented onset and decline in expression of several regulatory genes through aging [39] combined with these results in enucleated mice supports our hypothesis that activity modifiable gene expression may be a guiding force in the establishment of critical period limits of inter-area plasticity in the neocortex. Future studies that isolate the removal of different types of retinal activity, such as through the use of chemicals that reduce or eliminate ganglion cell activity or dark rearing, are needed to further our investigation into how activity-dependent features (such as sensory input) and activity-independent mechanisms (such as gene expression) interact to partition the neocortex into functional sensory and motor areas in development and how areal features and subdivisions are maintained throughout the life of the animal.

## Methods

### Mouse colony

All breeding and experimental studies were conducted in strict accordance with protocol guidelines approved by the Institutional Animal Care and Use Committee at the University of California, Riverside. All mice were maintained in a CD1 background and originally purchased from The Jackson Laboratory, Bar Harbor, Maine, USA.

### Newborn bilateral enucleation

All enucleation procedures were conducted on newborn mice on P0; newborns were allowed to nurse briefly after birth (1 hour) prior to surgery. Pups were initially anesthetized with an intraperitoneal injection of a ketamine (40 mg/kg) and xylazine (5 mg/kg) cocktail and were placed on briefly ice [94,95] before the removal of each eye (1 to 4 minutes). After a surgical level of anesthesia was achieved and verified with a toe-pinch, the eyelid was opened with a scalpel. The eye was lifted away from the orbit with forceps and freed from the surrounding musculature and optic nerve using surgical scissors. Following eye removal, the eyelid was closed and sealed using 0.5 μL to 1 μL of tissue adhesive (Surgi-Lock instant liquid tissue adhesive, Fisher Scientific, Pittsburgh, PA, USA). Pups were revived by partial immersion in a lukewarm water bath for 30 seconds and dried. A thin coat of Lidocaine hydrochloride jelly USP, 2% (Akorn, Lake Forest, IL, USA and erythromycin ophthalmic ointment USP, 0.5% (Bausch & Lomb, Rochester, NY, USA) was applied post-surgery to prevent pain and infection. Newborn control pups were subjected to anesthesia and revival procedures as described above. The total time allowed for aseptic surgical procedures within one litter was no greater than 2 hours to promote standardization between litters. Pups were housed with their mother in a cage with nesting material before and after surgery.

### Tissue preparation

All control and experimental animals were euthanized with a lethal dose of sodium pentobarbital (100 mg/kg) and transcardially perfused with 4% paraformaldehyde (PFA) in 0.1 M phosphate buffer (pH 7.4) at P10. In all cases, the brain was removed from the skull and hemisected, with one hemisphere used for postmortem tracing, and the opposite hemisphere used for *in situ *RNA hybridization. Hemispheres used for postmortem dye tracing were post-fixed with 4% PFA at room temperature during the 12 weeks required for tracer transport after dye-crystal placement. Hemispheres reserved for *in situ *RNA hybridization were post-fixed overnight at 4°C and then step-immersed into methanol for dehydration, and stored at -20°C. Additional hemispheres were obtained for Nissl staining.

### Gene expression assays

Gene expression assays were conducted using standard protocols and methods for non-radioactive free-floating *in situ *RNA hybridization [6,28,39,96,97]. The following probes were used to identify the patterns of neocortical gene expression at P10 (see Additional file 1 for full probe sequences): *ephrin A5, Id2, nuclear receptor subfamily 2, group F, member 1*, also known as *COUP-TFI *and *RZRß *(gifts from John Rubenstein, UCSF)*, Lhx2 *(a gift from Juan Botas, Baylor College of Medicine) and *Cadherin 8*, (*Cad8*, a gift from Masatoshi Takeichi, Riken Center for Developmental Biology, Japan). To prepare tissue for *in situ *RNA hybridization, hemispheres reserved for hybridization were rehydrated through a methanol series, embedded in gelatin-albumin and sectioned in the coronal or sagittal plane at 100 μm using a Vibratome. After hybridization, all sections were mounted in glycerol onto glass slides, covered by a cover slip and photographed as described below. Half of the hemispheres obtained from 14 successful P10 enucleated mice (seven hemispheres cut in the coronal plane, seven in the sagittal plane) were processed for ISH. Each hemisphere was processed for six genes to achieve at least seven replicates of each gene in each plane of section. Additionally, half of the hemispheres obtained from 10 P10 sham control mice (seven hemispheres cut in the coronal plane, seven in the sagittal plane) for each of six genes were processed for ISH and included in our study. These samples were used in the demonstration of raw data and the ImageJ density measurements.

### Anatomical tracing techniques

DiI and DiA (both Invitrogen, San Diego, CA, USA) crystals were placed in postmortem neocortical tissue to determine the patterns of ipsilateral INCs in wild-type control mice and bilaterally enucleated mice; this method has been used to describe mouse INCs in a diverse range of prenatal and postnatal ages [28,39], and has been described elsewhere in detail [6,10,98]. Single crystals of DiI and DiA were placed in two discrete areas in a single hemisphere of the neocortex: the parietal (somatosensory) cortex and occipital (visual) cortex. Each dye crystal was placed in a morphologically defined location, using a dye placement grid to enhance reliability of the crystal placement [6,28,39]. The location of somatosensory and visual cortical areas in the P10 mouse neocortex was determined and verified in our previous studies in normal mice [39]. As in prior studies, dye crystal placements were verified using thalamocortical labeling; for example a visual cortex occipital dye placement was verified only if retrogradely labeled cells were present in the LGN. As there were no effects on TCAs for either visual or somatosensory DPLs in the enucleated animals (see Figure 5), this method was utilized for both control and experimental mice. Note that in the figures and text, we refer to the caudal occipital DPLs in the mouse cortex as the visual cortex; this is done out of convention as we recognize that identifying this region as a visual area in the enucleated mouse may be misleading. After dye placement, brains were immersed in 4% PFA at room temperature for 12 weeks to allow for transport of the tracer. Prior to sectioning, the transport of dye to the thalamus was confirmed by examining the medial side of the hemisected injected brain under a fluorescent dissection microscope; if the retrograde tracer had reached the thalamic nuclei, the labeled internal capsule and thalamus was easily observed through the near-translucent tissue. This method is an effective way to optimize our dye transport times. All tissue was sectioned in the coronal plane at 100 μm using a Vibratome. Sections were immediately counter-stained with crystallized 4',6-diamidine-2-phenylindole dihydrochloride (DAPI; Roche, Nutley, NJ, USA), mounted onto glass slides, covered with a cover slip with Vectashield mounting medium for fluorescence (Vector Laboratories, Inc., Burlingame, CA, USA) and photographed as described below. We required at least seven replicates for each DPL in both enucleated and control brains. Because it was important to accurately detect the S-V boundary, the somatosensory and visual cortex DPLs were placed in the same hemisphere, and seven replicates of each area dye placement were assessed in both control (seven hemispheres with two DPLs each) and experimental (seven hemispheres with two DPLs each). These samples were used for the presentation of raw data, reconstruction of the lateral view two-dimensional drawings and the micrometer measurements used for the statistical assessments of the medial shift of somatosensory INCs.

### Nissl Staining

Five hemispheres each from five P10 enucleated mice and five P10 controls were cryoprotected post-perfusion using 40% sucrose in 0.1 M phosphate buffer for three days at 4°C and sectioned using a cryostat at 40 μm. The sections were mounted on glass slides, allowed to dry overnight, stained for Nissl substance and coverslipped with Permount mounting media (Fisher Scientific, Pittsburgh, PA, USA).

### Analysis of gene expression assays, Nissl staining and dye tracing

All images were captured by a digital high resolution Zeiss Axio camera using Axiovision software (Version 4.7). Sections processed for *in situ *RNA hybridization or Nissl staining were digitally imaged using bright field on a Zeiss Stereo Discovery V12 stereomicroscope. The staining achieved from this specific method of ISH was used as a qualitative tool to determine the position of expression in the P10 mouse neocortex, and subsequently measured quantitatively (see below). We present images of enucleated brain tissue where the specific locations of expression can be clearly determined, in representative cases, aligned side-by-side with control sections at the exact or near-exact anatomical level. We examined multiple cases to ensure that any observed changes in the position of expression are a true and reliable phenotype of our treatment, enucleation. This method has been used previously to determine the effects of treatment or mutation [6,10,98] and is a standard method of analysis in the field of developmental neurobiology in multiple species. Nissl sections were used to aid in the definition of thalamic nuclear boundaries and cortical lamina. For the analysis of dye tracing experiments, all sections were digitally imaged three times by a Zeiss Axio Imager Upright Microscope equipped with fluorescence. The three filters used were as follows: blue for DAPI counterstain, red for DiI and green for DiA labeling. (Excitation wavelengths - blue: DAPI, 359 nm; red: Cyanine 3, 550 nm; green: GFP, 470 nm. Emission wavelengths - blue: DAPI, 461 nm; red: Cyanine 3, 570 nm; green: GFP, 509 nm.) Captured images were then merged and saved in high-resolution format for analysis. In order to visualize the INCs in a lateral view, the previously published method of INC flattening was utilized, where all sections were drawn in National Institutes of Health image software, including anatomical structures, DPLs and cell bodies. The section drawings are stacked, aligned and morphed into a lateral view illustration. For detailed methods, please see [28,39]. Lateral view reconstructions of P10 control and enucleated neocortices, which demonstrate the location and spread of DPLs and associated retrogradely labeled cells, are illustrated in Figure 4. All tracing cases were flattened using this method and illustrations matching the cases shown in Figure 4 were presented. Green shapes and dots in the reconstructions represent DPLs and cells from somatosensory dye placements, while red shapes and dots represent DPLs and cells from the visual cortex dye placements.

### Quantification

Following ISH procedures and digital imaging of sections, cortical expression of *ephrin A5 *was quantified using ImageJ, with emphasis placed on the analysis of ectopic medial cortical expression present near the natural S-V boundary. First, the digital images were converted to a binary form and a threshold was defined, with identical anatomic levels being maintained across all replicates (n = 7 control and n = 7 enucleates). An ROI1 was then defined by electronically positioning a grid measuring 750 μm × 600 μm over the medial parietal cortex. A second area of equivalent dimension immediately lateral to ROI1 was also defined, serving as an internal control (ROI2). Each area was then individually measured to determine the amount of expression in the particular region. The total expression value was recorded as percent of tissue (area fraction) expressing the selected transcript within the ROI.

To quantify the observation of a shift in somatosensory INCs at the S-V boundary, we measured the position of the most medial labeled cell resulting from a somatosensory dye placement, as the distance from the cortical midline edge using a high-precision micrometer within the Zeiss Axiovision software program. All cases included in these analyses had somatosensory DPLs that were precisely and reliably placed using a dye placement grid [17] (n = 7 control and n = 7 enucleates).

Values for ISH density measures and INC distances from midline are reported as the mean ± standard error. The mean for each control group was established from the seven measurements in control tissue, and the mean for each enucleated group was established from the seven measurements in enucleated tissue. Two-sample t-tests were used to compare gene expression density in the two ROIs and the distance of dye labeled cells from the medial cortical border across control and enucleated groups. A *P*-value of less than 0.05 was chosen for statistical significance between groups.

## Abbreviations

APP: areal patterning period; *Cad8*: *Cadherin-8*; *COUP-TFI*: *chicken ovalbumin upstream promoter transcription factor 1*; DAPI: 4',6-diamidine-2-phenylindole dihydrochloride; dLGN: dorsal lateral geniculate nucleus; DiA: 4-(4-(dihexadecylamino)styryl)-N-methylpyridinium iodide; DiI: 1,1'-dioctadecyl-3,3,3',3'-tetramethylindocarbocyanine perchlorate; DPL: dye placement location; E: embryonic day; *Id2*: *inhibitor of DNA binding 2*; GFP: green fluorescent protein; INC: ipsilateral intraneocortical connection; ISH: *in situ *RNA hybridization; LGN: lateral geniculate nucleus; *Lhx2*: *LIM homeobox protein 2*; PFA: paraformaldehyde; P: postnatal day; ROI: region of interest; *RZRß*: *retinoic acid receptor related orphan receptor beta*; S-V: somatosensory-visual; TCA: thalamocortical afferents; VPN: ventral posterior nucleus.

## Competing interests

The authors declare that they have no competing interests.

## Authors' contributions

CAD carried out day of birth enucleations and ISH experiments and helped draft the manuscript. CWA performed dye-tracing experiments, conducted quantitative analyses, and helped with figure preparation and references. KJH designed the experiments, performed data analysis, prepared the figures and drafted the manuscript. All authors read and approved the final manuscript.

## Supplementary Material

Additional file 1 ** RNA probe sequences**. cDNA Sequences Utilized To Generate Digoxigenin-labeled RNA Probes. Click here for file

## References

[B1] Rakic P Specification of cerebral cortical areas Science 1988 241 170 176 10.1126/science.3291116 3291116

[B2] O'Leary DD Do cortical areas emerge from a protocortex? Trends Neurosci 1989 12 10 400 406 10.1016/0166-2236(89)90080-5 2479138

[B3] Rubenstein JL Anderson S Shi L Miyashita-Lin E Bulfone A Hevner R Genetic control of cortical regionalization and connectivity Cereb Cortex 1999 9 524 532 10.1093/cercor/9.6.524 10498270

[B4] Miyashita-Lin E Hevner R Wassarman KM Martinez S Martin GR Rubenstein JL Early neocortical regionalization in the absence of thalamic innervation Science 1999 285 906 909 10.1126/science.285.5429.906 10436162

[B5] Nakagawa Y Johnson JE O'Leary DD Graded and areal expression patterns of regulatory genes and cadherins in embryonic neocortex independent of thalamocortical input J Neurosci 1999 9 10877 10885 10594069 10.1523/JNEUROSCI.19-24-10877.1999 PMC6784968

[B6] Huffman KJ Garel S Rubenstein JL Fgf8 regulates the development of intra-neocortical projections J Neurosci 2004 24 8917 8923 10.1523/JNEUROSCI.2086-04.2004 15483110 PMC6730060

[B7] Nakagawa Y O'Leary DD Dynamic patterned expression of orphan nuclear receptor genes RORalpha and RORbeta in developing mouse forebrain Dev Neurosci 2003 25 2-4 234 244 10.1159/000072271 12966220

[B8] O'Leary DD Chou SJ Sahara S Area patterning of the mammalian cortex Neuron 2007 56 2 252 269 10.1016/j.neuron.2007.10.010 17964244

[B9] Zhou X Suh J Cerretti DP Zhou R DiCicco-Bloom E Ephrins stimulate neurite outgrowth during early cortical neurogenesis J Neurosci Res 2001 66 6 1054 1063 10.1002/jnr.10029 11746437

[B10] Garel S Huffman KJ Martin G Rubenstein JL Molecular regionalization of the neocortex is disrupted in Fgf8 hypomorphic mutants Development 2003 130 1903 1914 10.1242/dev.00416 12642494

[B11] Donoghue MJ Rakic P Molecular evidence for the early specification of presumptive functional domains in the embryonic primate cerebral cortex J Neurosci 1999 19 14 5967 5979 10407035 10.1523/JNEUROSCI.19-14-05967.1999 PMC6783094

[B12] Bishop K Goudreau G O'Leary DD Regulation of area identity in the mammalian neocortex by Emx2 and Pax6 Science 2000 288 344 349 10.1126/science.288.5464.344 10764649

[B13] Liu Q Dwyer ND O'Leary DD Differential expression of COUP-TFI, CHL1, and two novel genes in developing neocortex identified by differential display PCR J Neurosci 2000 20 20 7682 7690 11027229 10.1523/JNEUROSCI.20-20-07682.2000 PMC6772850

[B14] Ragsdale CW Grove EA Patterning the mammalian cerebral cortex Curr Opin Neurobiol 2001 11 50 58 10.1016/S0959-4388(00)00173-2 11179872

[B15] Ruiz I Altaba A Gitton Y Dahmane N Embryonic regionalization of the neocortex Mech Dev 2001 107 3 11 10.1016/S0925-4773(01)00422-1 11520659

[B16] Cecchi C Emx2: a gene responsible for cortical development, regionalization and area specification Gene 2002 291 1 9 10.1016/S0378-1119(02)00623-6 12095673

[B17] Yun ME Johnson RR Antic A Donoghue MJ EphA family gene expression in the developing mouse neocortex: regional patterns reveal intrinsic programs and extrinsic influence J Comp Neurol 2003 456 203 216 10.1002/cne.10498 12528186

[B18] Fukuchi-Shimogori T Grove EA Emx2 patterns the neocortex by regulating FGF positional signaling Nat Neurosci 2003 6 825 831 10.1038/nn1093 12872126

[B19] Shimogori T Banuchi V Ng HY Strauss JB Grove EA Embryonic signaling centers expressing BMP, WNT and FGF proteins interact to pattern the cerebral cortex Development 2004 131 5639 5647 10.1242/dev.01428 15509764

[B20] Abu-Khalil A Fu L Grove EA Zecevic N Geschwind DH Wnt genes define distinct boundaries in the developing human brain: implications for human forebrain patterning J Comp Neurol 2004 474 276 288 10.1002/cne.20112 15164427

[B21] Funatsu N Inoue T Nakamura S Gene expression analysis of the late embryonic mouse cerebral cortex using DNA microarray: identification of several region- and layer-specific genes Cereb Cortex 2004 14 1031 1044 10.1093/cercor/bhh063 15142957

[B22] Hamasaki T Leingartner A Ringstedt T O'Leary DD EMX2 regulates sizes and positioning of the primary sensory and motor areas in neocortex by direct specification of cortical progenitors Neuron 2004 43 359 372 10.1016/j.neuron.2004.07.016 15294144

[B23] Sansom SN Hebert JM Thammongkol U Smith J Nisbet G Surani MA McConnell SK Livesey FJ Genomic characterisation of a Fgf- regulated gradient-based neocortical protomap Development 2005 132 3947 3961 10.1242/dev.01968 16079153 PMC4729368

[B24] Mallamaci A Stoykova A Gene networks controlling early cerebral cortex arealization Eur J Neurosci 2006 23 847 856 10.1111/j.1460-9568.2006.04634.x 16519650

[B25] Cholfin JA Rubenstein JL Frontal cortex subdivision patterning is coordinately regulated by Fgf8, Fgf17, and Emx2 J Comp Neurol 2008 509 144 155 10.1002/cne.21709 18459137 PMC4399554

[B26] O'Leary DD Sahara S Genetic regulation of arealization of the neocortex Curr Opin Neurobiol 2008 18 90 100 10.1016/j.conb.2008.05.011 18524571 PMC2677555

[B27] Rakic P Ayoub AE Breunig JJ Dominguez MH Decision by division: making cortical maps Trends Neurosci 2009 32 291 301 10.1016/j.tins.2009.01.007 19380167 PMC3601545

[B28] Dye CA El Shawa H Huffman KJ A lifespan analysis of intraneocortical connections and gene expression in the mouse I Cereb Cortex 2011 21 6 1311 1330 10.1093/cercor/bhq212 21060110 PMC3140180

[B29] Zhou L Gall D Qu Y Prigogine C Cheron G Tissir F Schiffmann SN Goffinet AM Maturation of "neocortex isole" *in vivo *in mice J Neurosci 2010 30 23 7928 7939 10.1523/JNEUROSCI.6005-09.2010 20534841 PMC6632698

[B30] Kahn DM Krubitzer LA Massive cross-modal cortical plasticity and the emergence of a new cortical field in developmentally blind mammals Proc Natl Acad Sci USA 2002 99 11429 11434 10.1073/pnas.162342799 12163645 PMC123273

[B31] Karlen SJ Kahn DM Krubitzer L Early blindness results in abnormal corticocortical and thalamocortical connections Neurosci 2006 142 3 843 858 10.1016/j.neuroscience.2006.06.055 16934941

[B32] Karlen SJ Krubitzer L Effects of bilateral enucleation on the size of visual and nonvisual areas of the brain Cereb Cortex 2009 19 6 1360 1371 10.1093/cercor/bhn176 18842663 PMC2677651

[B33] Campi KL Bales KL Grunewald R Krubitzer L Connections of auditory and visual cortex in the prairie vole (*Microtus ochrogaster*): evidence for multisensory processing in primary sensory areas Cereb Cortex 2010 20 1 89 108 10.1093/cercor/bhp082 19395525 PMC2792189

[B34] Mooney R Penn AA Gallego R Shatz CJ Thalamic relay of spontaneous retinal activity prior to vision Neuron 1996 17 5 863 874 10.1016/S0896-6273(00)80218-4 8938119

[B35] Weliky M Katz LC Correlational structure of spontaneous neuronal activity in the developing lateral geniculate nucleus *in vivo* Science 1999 285 5427 599 604 10.1126/science.285.5427.599 10417392

[B36] Huberman AD Feller MB Chapman B Mechanisms underlying development of visual maps and receptive fields Annu Rev Neurosci 2008 31 470 509 10.1146/annurev.neuro.31.060407.125533 PMC2655105 18558864

[B37] Molnár Z Adams R Goffinet AM Blakemore C The role of the first postmitotic cortical cells in the development of thalamocortical innervation in the reeler mouse J Neurosci 1998 18 15 5746 5765 9671664 10.1523/JNEUROSCI.18-15-05746.1998 PMC6793036

[B38] Bansal A Singer JH Hwang BJ Xu W Beaudet A Feller MB Mice lacking specific nicotinic acetylcholine receptor subunits exhibit dramatically altered spontaneous activity patterns and reveal a limited role for retinal waves in forming ON and OFF circuits in the inner retina J Neurosci 2000 20 20 7672 7681 11027228 10.1523/JNEUROSCI.20-20-07672.2000 PMC6772851

[B39] Dye CA El Shawa H Huffman KJ A lifespan analysis of intraneocortical connections and gene expression in the mouse II Cereb Cortex 2011 21 6 1331 1350 10.1093/cercor/bhq213 21060113 PMC3140181

[B40] Armentano M Chou SJ Tomassy GS Leingärtner A O'Leary DD Studer M COUP-TFI regulates the balance of cortical patterning between frontal/motor and sensory areas Nat Neurosci 1999 10 1277 1286 17828260 10.1038/nn1958

[B41] Faedo A Tomassy GS Ruan Y Teichmann H Krauss S Pleasure SJ Tsai SY Tsai MJ Studer M Rubenstein JL COUP-TFI coordinates cortical patterning, neurogenesis, and laminar fate and modulates MAPK/ERK, AKT, and beta-catenin signaling Cereb Cortex 2008 18 9 2117 2131 18165280 10.1093/cercor/bhm238 PMC2733307

[B42] Tomassy GS de Leonibus E Jabaudon D Lodato S Alfano C Mele A Macklis JD Studer M Area-specific temporal control of corticospinal motor neuron differentiation by COUP-TFI Proc Natl Acad Sci USA 2010 107 8 3576 3581 10.1073/pnas.0911792107 20133588 PMC2840488

[B43] Grove EA Fukuchi-Shimogori T Generating the cerebral cortical area map Annu Rev Neurosci 2003 26 355 380 10.1146/annurev.neuro.26.041002.131137 14527269

[B44] Sur M Rubenstein JL Patterning and plasticity of the cerebral cortex Science 2005 310 805 810 10.1126/science.1112070 16272112

[B45] Mizuno H Hirano T Tagawa Y Evidence for activity-dependent cortical wiring: formation of interhemispheric connections in neonatal mouse visual cortex requires projection neuron activity J Neurosci 2007 27 25 6760 6770 10.1523/JNEUROSCI.1215-07.2007 17581963 PMC6672694

[B46] Larsen DD Luu JD Burns ME Krubitzer L What are the effects of severe visual impairment on the cortical organization and connectivity of primary visual cortex? Front Neuroanat 2009 3 30 20057935 10.3389/neuro.05.030.2009 PMC2802552

[B47] Majdan M Shatz CJ Effects of visual experience on activity-dependent gene regulation in cortex Nat Neurosci 2006 5 650 659 16582906 10.1038/nn1674

[B48] Desgent S Boire D Ptito M Altered expression of parvalbumin and calbindin in interneurons within the primary visual cortex of neonatal enucleated hamsters Neuroscience 2010 4 1326 1340 20937364 10.1016/j.neuroscience.2010.10.016

[B49] Goldshmit Y Galley S Foo D Sernagor E Bourne JA Anatomical changes in the primary visual cortex of the congenitally blind Crx-/- mouse Neurosci 2010 166 3 886 898 10.1016/j.neuroscience.2009.12.039 20034544

[B50] Bock AS Olavarria JF Leigland LA Taber EN Jespersen SN Kroenke CD Diffusion tensor imaging detects early cerebral cortex abnormalities in neuronal architecture induced by bilateral neonatal enucleation: an experimental model in the ferret Front Syst Neurosci 2010 4 149 21048904 10.3389/fnsys.2010.00149 PMC2971465

[B51] Suzuki SC Inoue T Kimura Y Tanaka T Takeichi M Neuronal circuits are subdivided by differential expression of type-II classic cadherins in postnatal mouse brains Mol Cell Neurosci 1997 9 5-6 433 447 10.1006/mcne.1997.0626 9361280

[B52] Mackarehtschian K Lau CK Caras I McConnell SK Regional differences in the developing cerebral cortex revealed by ephrin-A5 expression Cereb Cortex 1999 9 6 601 610 10.1093/cercor/9.6.601 10498278

[B53] Vanderhaeghen P Lu Q Prakash N Frisén J Walsh CA Frostig R Flanagan JG A mapping label required for normal scale of body representation in the cortex Nat Neurosci 2000 3 358 365 10.1038/73929 10725925

[B54] Bishop KM Rubenstein JL O'Leary DD Distinct actions of Emx1, Emx2, and Pax6 in regulating the specification of areas in the developing neocortex J Neurosci 2002 22 17 7627 7638 12196586 10.1523/JNEUROSCI.22-17-07627.2002 PMC6757966

[B55] Chou SJ Perez-Garcia CG Kroll TT O'Leary DD Lhx2 specifies regional fate in Emx1 lineage of telencephalic progenitors generating cerebral cortex Nat Neurosci 2009 12 11 1381 1389 10.1038/nn.2427 19820705 PMC2897740

[B56] Blakemore C Sensitive and vulnerable periods in the development of the visual system Ciba Found Symp 1991 156 129 147 1855408 10.1002/9780470514047.ch9

[B57] Wiesel TN Hubel DH Effects of visual deprivation on morphology and physiology of cells in the cats lateral geniculate body J Neurophysiol 1963 26 978 993 14084170 10.1152/jn.1963.26.6.978

[B58] Wiesel TN Hubel DH Single-cell responses in striate cortex of kittens deprived of vision in one eye J Neurophysiol 1963 26 1003 1017 14084161 10.1152/jn.1963.26.6.1003

[B59] Feldheim DA Kim YI Bergemann AD Frisén J Barbacid M Flanagan JG Genetic analysis of ephrin-A2 and ephrin-A5 shows their requirement in multiple aspects of retinocollicular mapping Neuron 2000 25 3 563 574 10.1016/S0896-6273(00)81060-0 10774725

[B60] Dehay C Giroud P Berland M Killackey H Kennedy H Contribution of thalamic input to the specification of cytoarchitectonic cortical fields in the primate: effects of bilateral enucleation in the fetal monkey on the boundaries, dimensions, and gyrification of striate and extrastriate cortex J Comp Neurol 1996 367 1 70 89 10.1002/(SICI)1096-9861(19960325)367:1<70::AID-CNE6>3.0.CO;2-G 8867284

[B61] Asanuma C Stanfield BB Induction of somatic sensory inputs to the lateral geniculate nucleus in congenitally blind mice and in phenotypically normal mice Neurosci 1990 39 3 533 545 10.1016/0306-4522(90)90241-U 1711167

[B62] Heumann D Rabinowicz T Postnatal development of the dorsal lateral geniculate nucleus in the normal and enucleated albino mouse Exp Brain Res 1980 38 1 75 85 7351229 10.1007/BF00237933

[B63] Warton SS Dyson SE Harvey AR Visual thalamocortical projections in normal and enucleated rats: HRP and fluorescent dye studies Exp Neurol 1988 100 1 23 39 10.1016/0014-4886(88)90198-7 3350091

[B64] Izraeli R Koay G Lamish M Heicklen-Klein AJ Heffner HE Heffner RS Wollberg Z Cross-modal neuroplasticity in neonatally enucleated hamsters: structure, electrophysiology and behaviour Eur J Neurosci 2002 15 693 712 10.1046/j.1460-9568.2002.01902.x 11886450

[B65] Williams AL Reese BE Jeffery G Role of retinal afferents in regulating growth and shape of the lateral geniculate nucleus J Comp Neurol 2002 445 3 269 277 10.1002/cne.10171 11920706

[B66] Cang J Kaneko M Yamada J Woods G Stryker MP Feldheim DA Ephrin-as guide the formation of functional maps in the visual cortex Neuron 2005 48 4 577 589 10.1016/j.neuron.2005.10.026 16301175 PMC2424263

[B67] Krubitzer L Huffman KJ Arealization of the neocortex in mammals: genetic and epigenetic contributions to the phenotype Brain Behav Evol 2000 55 6 322 335 10.1159/000006667 10971017

[B68] Pallas SL Intrinsic and extrinsic factors that shape neocortical specification Trends Neurosci 2001 24 7 417 423 10.1016/S0166-2236(00)01853-1 11410273

[B69] Lyckman AW Sur M Role of afferent activity in the development of cortical specification Results Probl Cell Differ 2002 39 139 156 12353467 10.1007/978-3-540-46006-0_7

[B70] Molnár Z Higashi S López-Bendito G Choreography of early thalamocortical development Cereb Cortex 2003 13 6 661 669 10.1093/cercor/13.6.661 12764042

[B71] Cullen MJ Kaiserman-Abramof IR Cytological organization of the dorsal lateral geniculate nuclei in mutant anophthalmic and postnatally enucleated mice J Neurocytol 1976 5 4 407 424 10.1007/BF01181648 993820

[B72] Robertson RT Poon HK Duran MR Yu J Neonatal enucleations reduce number, size, and acetylcholinesterase histochemical staining of neurons in the dorsal lateral geniculate nucleus of developing rats Brain Res Dev Brain Res 1989 47 2 209 225 10.1016/0165-3806(89)90177-6 2743558

[B73] Grubb MS Rossi FM Changeux JP Thompson ID Abnormal functional organization in the dorsal lateral geniculate nucleus of mice lacking the beta 2 subunit of the nicotinic acetylcholine receptor Neuron 2003 40 6 1161 1172 10.1016/S0896-6273(03)00789-X 14687550

[B74] Akaneya Y Sohya K Kitamura A Kimura F Washburn C Zhou R Ninan I Tsumoto T Ziff EB Ephrin-A5 and EphA5 interaction induces synaptogenesis during early hippocampal development PLoS One 2010 5 8 e12486 10.1371/journal.pone.0012486 20824214 PMC2930854

[B75] Hara Y Nomura T Yoshizaki K Frisén J Osumi N Impaired hippocampal neurogenesis and vascular formation in ephrin-A5-deficient mice Stem Cells 2010 28 5 974 983 20474079 10.1002/stem.427

[B76] Pfeiffenberger C Yamada J Feldheim DA Ephrin-As and patterned retinal activity act together in the development of topographic maps in the primary visual system J Neurosci 2006 26 50 12873 12884 10.1523/JNEUROSCI.3595-06.2006 17167078 PMC3664553

[B77] Lasorella A Stegmüller J Guardavaccaro D Liu G Carro MS Rothschild G de la Torre-Ubieta L Pagano M Bonni A Iavarone A Degradation of Id2 by the anaphase-promoting complex couples cell cycle exit and axonal growth Nature 2006 442 7101 471 474 10.1038/nature04895 16810178

[B78] Suzuki SC Furue H Koga K Jiang N Nohmi M Shimazaki Y Katoh-Fukui Y Yokoyama M Yoshimura M Takeichi M Cadherin-8 is required for the first relay synapses to receive functional inputs from primary sensory afferents for cold sensation J Neurosci 2007 27 13 3466 3476 10.1523/JNEUROSCI.0243-07.2007 17392463 PMC6672125

[B79] Cynader M Prolonged sensitivity to monocular deprivation in dark-reared cats: effects of age and visual exposure Brain Res 1983 284 2-3 155 164 6871721 10.1016/0165-3806(83)90002-0

[B80] Gordon JA Stryker MP Experience-dependent plasticity of binocular responses in the primary visual cortex of the mouse J Neurosci 1996 16 10 3274 3286 8627365 10.1523/JNEUROSCI.16-10-03274.1996 PMC6579137

[B81] Antonini A Fagiolini M Stryker MP Anatomical correlates of functional plasticity in mouse visual cortex J Neurosci 1999 19 11 4388 4406 10341241 10.1523/JNEUROSCI.19-11-04388.1999 PMC2452998

[B82] Nedivi E Molecular analysis of developmental plasticity in neocortex J Neurobiol 1999 41 1 135 147 10.1002/(SICI)1097-4695(199910)41:1<135::AID-NEU17>3.0.CO;2-F 10504201 PMC3062904

[B83] Dahlhaus M Wan Li K van der Schors RC Saiepour MH van Nierop P Heimel JA Hermans JM Loos M Smit AB Levelt CN The synaptic proteome during development and plasticity of the mouse visual cortex Mol Cell Proteomics 2011 10 5 M110.005413 10.1074/mcp.M110.005413 21398567 PMC3098591

[B84] Hanganu IL Ben-Ari Y Khazipov R Retinal waves trigger spindle bursts in the neonatal rat visual cortex J Neurosci 2006 26 25 6728 6736 10.1523/JNEUROSCI.0752-06.2006 16793880 PMC6673818

[B85] Colonnese MT Khazipov R "Slow activity transients" in infant rat visual cortex: a spreading synchronous oscillation patterned by retinal waves J Neurosci 2010 30 12 4325 4337 10.1523/JNEUROSCI.4995-09.2010 20335468 PMC3467103

[B86] Calford MB Dynamic representational plasticity in sensory cortex Neurosci 2002 111 4 709 738 10.1016/S0306-4522(02)00022-2 12031401

[B87] Meister M Wong RO Baylor DA Shatz CJ Synchronous bursts of action potentials in ganglion cells of the developing mammalian retina Science 1991 252 5008 939 943 10.1126/science.2035024 2035024

[B88] Krug K Akerman CJ Thompson ID Responses of neurons in neonatal cortex and thalamus to patterned visual stimulation through the naturally closed lids J Neurophysiol 2001 85 4 1436 1443 11287467 10.1152/jn.2001.85.4.1436

[B89] Mrsic-Flogel TD Hofer SB Creutzfeldt C Cloëz-Tayarani I Changeux JP Bonhoeffer T Hübener M Altered map of visual space in the superior colliculus of mice lacking early retinal waves J Neurosci 2005 25 29 6921 6928 10.1523/JNEUROSCI.1555-05.2005 16033902 PMC6725344

[B90] Dütting D Handwerker C Drescher U Topographic targeting and pathfinding errors of retinal axons following overexpression of ephrinA ligands on retinal ganglion cell axons Dev Biol 1999 216 1 297 311 10.1006/dbio.1999.9489 10588880

[B91] Yates PA Roskies AL McLaughlin T O'Leary DD Topographic-specific axon branching controlled by ephrin-As is the critical event in retinotectal map development J Neurosci 2001 21 21 8548 8563 11606643 10.1523/JNEUROSCI.21-21-08548.2001 PMC6762786

[B92] Hanson MG Landmesser LT Normal patterns of spontaneous activity are required for correct motor axon guidance and the expression of specific guidance molecules Neuron 2004 43 5 687 701 10.1016/j.neuron.2004.08.018 15339650

[B93] Huberman AD Murray KD Warland DK Feldheim DA Chapman B Ephrin-As mediate targeting of eye-specific projections to the lateral geniculate nucleus Nat Neurosci 2005 8 8 1013 1021 10.1038/nn1505 16025110 PMC2652399

[B94] Robertson RT Fogolin RP Tijerina AA Yu J Effects of neonatal monocular and binocular enucleation on transient acetylcholinesterase activity in developing rat visual cortex Brain Res 1987 430 2 185 197 3607512 10.1016/0165-3806(87)90152-0

[B95] Zacharaki T Sophou S Giannakopoulou A Dinopoulos A Antonopoulos J Parnavelas JG Dori I Natural and lesion-induced apoptosis in the dorsal lateral geniculate nucleus during development Brain Res 2010 1344 62 76 20471376 10.1016/j.brainres.2010.05.021

[B96] Shimamura K Hirano S McMahon AP Takeichi M Wnt-1-dependent regulation of local E-cadherin and alpha N catenin expression in the embryonic mouse brain Development 1994 120 2225 2234 7925023 10.1242/dev.120.8.2225

[B97] Garel S Marìn F Grosschedl R Charnay P Ebf1 controls early cell differentiation in the embryonic striatum Development 1999 126 5285 5294 10556054 10.1242/dev.126.23.5285

[B98] Godement P Vanselow J Thanos S Bonhoeffer F A study in developing visual systems with a new method of staining neurones and their processes in fixed tissue Development 1987 101 697 713 2460302 10.1242/dev.101.4.697

