# Supplementary material for: Bilateral enucleation alters gene expression and intraneocortical connections in the mouse
Source: Neural Dev. 2012 Jan 30;7:5. doi: 10.1186/1749-8104-7-5 (PMC3347983; doi:10.1186/1749-8104-7-5)
Supplement: Additional file 1 — RNA probe sequences. cDNA Sequences Utilized To Generate Digoxigenin-labeled RNA Probes. [file 1749-8104-7-5-S1.PDF]

cDNA Sequences Utilized To Generate Digoxigenin-labeled RNA Probes

RZRß
GGCCCCCGAGGCGTGGCATTTTTTTTTTTTAAAGCAAGCACATTGGAGAGAAAGAAAAAAGA
AACAAACAAACAAACAAAAGCAAAACAAAACCCAGGCACCAGCCAGCTAGGACATTTTTTTT
CCACACTGCCTGAAAACAAACAAACAGCCATCAGAAACAGTCATCAGCAACAGCATCAAAAC
TGTTAGCTTAGCGGCGGCGGCCACCGTCACCCTGTGGCCACGACATCTGCCTAAAGGGATG
CTTCTCTCAGCGGAGCAGTTCTACGCCGACCGCCTTCTTCCCTCGTGCTGAGCGGGATTTTT
GGGTTCTCTGGGGTTCGGGCTGGGAGCTTCATGACTACACGGAGCAGGACAGCGGCCACAT
CATGCGAGCACAAATTGAAGTGATACCATGCAAAATTTGTGGCGATAAATCCTCCGGGATCC
ACTACGGAGTCATCACGTGTGAAGGCTGCAAGGGATTCTTCAGGAGGAGCCAGCAGAACAA
TGCCTCTTACTCCTGCCCAAGGCAGAGAAACTGTTTAATTGACAGAACCAACAGGAACCGTT
GCCAACACTGCCGCCTGCAGAAGTGTCTTGCCCTAGGAATGTCAAGAGATGCTGTGAAGTTT
GGGAGGATGTCCAAGAAGCAGCGGGACAGCCTGTATGCTGAGGTGCAGAAGCATCAGCAGA
GGCTGCAGGAGCAGCGGCAGCAGCAGAGTGGGGAGGCAGAGGCCCTTGCCAGGGTGTACA
GCAGCAGCATTAGCAATGGCCTCAGCAACCTGAACACCGAGACCGGCGGCACATACGCCAA
CGGGCACGTCATTGACCTGCCCAAGTCCGAGGGTTACTACAACATAGATTCCGGTCAACCGT
CTCCCGATCAGTCAGGACTGGACATGACTGGAATCAAACAGATAAAGCAAGAACCTATCTAT
GACCTCACATCCGTACACAACTTGTTTACCTATAGCTCTTTCAACAACGGGCAGTTAGCTCCC
GGGATAACAATGTCTGAGATCGATCGAATTGCACAGAACATCATTAAGTCCCATTTGGAGAC
GTGCCAGTACACAATGGAAGAGCTCCATCAGCTGGCATGGCAGACCCACACCTATGAGGAA
ATAAAGGCATATCAAAGCAAGTCCAGGGAGGCTCTGTGGCAGCAGTGTGCCATCCAGATCAC
CCACGCCATCCAATATGTGGTGGAGTTCGCAAAGCGGATAACAGGCTTCATGGAGCTGTGTC
AGAACGATCAGATCTTACTTCTGAAGTCAGGTTGCTTGGAAGTGGTTTTAGTGAGAATGTGCC
GTGCCTTCAACCCATTAAACAACACTGTTCTGTTTGAAGGAAAATATGGAGGAATGCAAATGT
TCAAAGCCTTAGGTTCTGATGACCTAGTGAATGAAGCATTTGACTTTGCGAAGAATCTGTGTT
CCTTGCAGCTGACCGAGGAAGAGATTGCTCTGTTCTCCTCTGCTGTTCTGATATCCCCAGAC
CGAGCCTGGCTGTTAGAACCAAGAAAAGTCCAGAAGCTTCAGGAAAAAATTTATTTTGCACTT
CAACATGTGATTCAGAAGAATCACCTGGATGATGAGACCCTGGCAAAGTTAATAGCCAAGAT
ACCAACTATCACGGCAGTCTGCAACTTGCATGGGGAGAAGCTACAGGTATTTAAGCAGTCTC
ATCCAGACATAGTGAATACACTGTTTCCTCCATTGTACAAGGAGCTCTTTAATCCTGACTGTG
CTGCGGTCTGCAAATGAAGGGGACGAGAACTCTCAGAGTCATGGAATGCATCGCCGTTAAG
ACAAAAGCAATGTGTTCATGGGGACTTAAGGAAAATGTCACTACTGCAACATTAGGAATGTCC
TGCACTTAATAGACATATTTTTCACCGCTACAGTTTGAAGAATGTAAATATGCACCTGAGTGG
GGCTCTTCTGTTTGTTCGTTTGTTGTTTGTTTGTTTTTGAAATGATCC

Lhx2
CGGTTCTCTGTGCAGCGCTGCGCCCGCTGCCACCTGGGCATCTCGGCCTCAGAGATGGTGATGCGCGC
TCGGGACTTGGTTTATCACCTCAACTGCTTCACATGCACAACGTGTAACAAGATGCTGACGACCGGCGAC
CATTTCGGCATGAAGGACAGCCTGGTCTATTGCCGCTTGCACTTCGAGGCTCTGCTGCAGGGCGAATAC
CCAGCACACTTTAACCATGCCGACGTGGCAGCGGCGGCAGCCGCAGCCGCAGCAGCTAAGAGTGCAGG
ATTGGGCTCAGCCGGGGCTAATCCGCTGGGTCTTCCCTACTACAACGGCGTGGGCACTGTGCAAAAGGG
GAGGCCGAGAAAGCGCAAGAGTCCAGGACCCGGGGCAGATCTGGCAGCTTACAACGCCGCGCTTAGCT
GTAACGAGAACGATGCTGAACACCTGGATCGTGACCAGCCCTACCCCAGCAGCCAAAAGACAAAGCGCA
TGCGCACCTCCTTCAAGCACCACCAGCTTCGGACAATGAAGTCTTACTTTGCCATTAACCACAATCCCGA
TGCCAAGGACTTGAAGCAGCTTGCGCAAAAGACCGGCCTCACCAAGAGAGTCCTCCAGGTCTGGTTTCA
GAATGCCCGGGCCAAGTTCAGGCGCAACCTTTTACG

Id2

TCTCATTCTGAACCGAGCCTGGTGCCGCGCAGTCAGCTCAGCCCCCTGTGGCGGCTCCCTCCCGGTCTT
CCTCCTACGAGCAGCATGAAAGCCTTCAGTCCGGTGAGGTCCGTTAGGAAAAACAGCCTGTCGGACCAC
AGCTTGGGCATCTCCCGGAGCAAAACCCCGGTGGACGACCCGATGAGTCTGCTCTACAACATGAACGAC
TGCTACTCCAAGCTCAAGGAACTGGTGCCCAGCATCCCCCAGAACAAGAAGGTGACCAAGATGGAAATC
CTGCAGCACGTCATCGATTACATCTTGGACCTGCAGATCGCCCTGGACTCGCATCCCACTATCGTCAGCC
TGCATCACCAGAGACCTGGACAGAACCAGGCGTCCAGGACGCCGCTGACCACCCTGAACACGGACATCA
GCATCCTGTCCTTGCAGGCATCTGAATTCCCTTCTGAGCTTATGTCGAATGATAGCAAAGTACTCTGTGG
CTAAATAAATGGCATTTGGGGACTTTTTTTTTTCTTTTTACTTTCTCTTTTTCTTTTGCACAAGAAGAAGTCT
ACAAGATCTTTTAAGACTTTTGTTATCAGCCATTTCACCAGGAGAACACGTTGAATGGACCTTTTTAAAAAG
AAAGCGGAAGGAAAACTAAGGATGATCGTCTTGCCCAGGTGTCGTTCTCCGGCCTGGACTGTGATACCG
TTATTTATGAGAGACTTTCAGTGCCCTTTCTACAGTTGGAAGGTTTTCTTTATATACTATTCCCACCATGGG
GAGCGAAAACGTTAAAAAAAAAAGAAAAAAATCACAAGGAATTGCCCAATGTAAGCAGACTTTGCCTTTTC
ACAAAGGTGGAGCGTGAATACCAGAAGGACCCAGTATTCGGTTACTTAAATGAAGTCTTCGGTCAGAAAT
GGCCTTTTTGACACGAGCCTACTGAATGCTGTGTATATATTTATATATAAATATATATATATTGAGTGAACC
TTGTGGACTCTTTAATTAGAGTTTTCTTGTATAGTGGCAGAAATAACCTATTTCTGCATTAAAATGTAATGA
CGTACTTATGCTAAACTTTTTATAAAAGTTTAGTTGTAAACTTAACCCTTTTATACAAAATAAA
TCAAGTGTGTTTATT

Cad8

ATGCCAGAAAGGCTAGCTGAGACGCTCATGGACCTCTGGACTCCATTAATAATATTATGGATTACCCTCC
CTTCTTGTGTATACACGGCTCCGATGAATCAGGCTCACGTTTTAACTACTGGATCCCCTTTGGAACTAAGC
AGGCAGAGTGAAGACATGCGGATTTTGAGCCGCTCCAAAAGAGGCTGGGTTTGGAATCAAATGTTTGTCC
TGGAAGAATTTTCTGGACCTGAACCGATTCTCGTTGGCCGGTTACACACAGATCTGGATCCTGGAAGCAA
AAAAATCAAGTATATCCTATCGGGTGATGGAGCTGGCACAATCTTTCAAATAAATGATATAACTGGAGATA
TCCATGCTATCAAAAGACTTGACCGAGAGGAAAAGGCTGAATATACGTTAACGGCTCAGGCAGTGGACTT
TGAGACAAACAAGCCCCTTGAGCCTCCTTCTGAATTTATTATTAAGGTTCAAGATATCAACGACAATGCAC
CTGAGTTCCTCAATGGACCTTATCATGCTACTGTTCCAGAGATGTCCATTTTGGGTACATCTGTCACTAAT
GTAACTGCCACTGATGCTGACGACCCAGTTTATGGAAACAGTGCAAAGTTGGTTTACAGTATCTTGGAGG
GACAGCCGTATTTTTCCATTGAGCCTGAAACAGCCATTATAAAAACTGCCCTTCCTAACATGGACAGAGAA
GCCAAGGAGGAATACCTGGTCGTAATACAAGCCAAAGATATGGGTGGACACTCTGGTGGTCTGTCTGGA
ACCACAACACTCACAGTGACCCTGACTGATGTTAATGACAATCCTCCAAAATTTGCTCAGAGTTTGTATCA
CTTCTCAGTACCAGAAGATGTGGTCCTTGGCACTGCGATAGGAAGGGTTAAGGCTAATGATCAGGATATT
GGTGAAAATGCACAATCATCCTATGACATCATTGATGGAGATGGGACAGCACTATTCGAAATCACTTCTGA
TGCCCAGGCCCAGGATGGTGTTATAAGACTGAGAAAGCCTCTGGACTTTGAGACCAAAAAATCCTATACT
CTGAAGGTGGAAGCAGCCAATATCCACATCGACCCACGTTTCAGTAGCAGGGGACCCTTTAAGGATACA
GCAACAGTCAAAATTGTCGTAGAGGATGCTGATGAGCCTCCGGTCTTCTCTTCACCAACTTACCTCCTTG
AAGTTCATGAAAATGCTGCTTTGAACTCTGTGATTGGCCAAGTGACAGCTCGTGACCCTGATATCACTTCC
AGTCCAATAAGGTTTTCCATTGACCGCCACACTGACCTGGAAAGACAGTTCAACATCAATGCAGATGATG
GGAAGATAACACTGGCAACTCCACTGGACAGAGAACTAAGTGTTTGGCACAACATCACCATCATTGCTAC
TGAGATCAGGAACCACAGTCAGATATCTCGAGTGCCTGTTGCTATTAAAGTGCTGGATGTCAATGACAAC
GCCCCTGAATTCGCATCCGAATATGAGGCATTTTTATGTGAAAATGGAAAACCCGGCCAAGTCATTCAAA
CAGTAAGTGCCATGGACAAAGACGATCCCAAAAACGGACATTTTTTCTTGTACAGTCTTCTTCCAGAAATG
GTCAACAACCCAAATTTCACCATCAAGAAAAATGAAGATAATTCCCTGAGCATTCTGGCAAAACATAATGG
ATTCAACCGCCAGAAGCAAGAAGTCTACCTTCTGCCTATAGTGATCAGTGACAGTGGAAACCCCCCTCTG
AGTAGCACCAGTACGTTGACCATCCGAGTCTGTGGCTGTAGCAATGATGGCGTGGTCCAGTCGTGCAAT
GTTGAAGCTTATGTCCTTCCTATTGGACTCAGTATGGGCGCCTTAATTGCTATATTAGCATGCATCATTTT
GCTGCTCGTCATTGTGGTTCTGTTTGTTACCCTGAGGAGGCACAAAAATGAACCACTTATTATCAAAGATG
ACGAAGACGTTCGAGAAAACATCATTCGCTACGACGACGAAGGAGGAGGGGAGGAGGACACAGAGGCT
TTTGACATTGCAACTTTGCAAAACCCAGATGGAATTAATGGATTTTTACCCCGGTGGGGATTGCCAACTCA
AAGGTGGGAGGAGAGAAATGCCATTCAAACAGGCTGTGTCTAG

EphA7

GTGGAGCAAGCGGCCGGTCTGCAGTCGGAGACTTGCAGGCAGCAAACACGGTGCGAACGAACCGGAGG
GGGGAGAGAGAAATCAAACAGCTAAGCGTGGAGCAGACGGCCTGGGACCCAGAAGGGGATCGATGCGA
GGAGCGCAATAATAACAACAATAATAACCCACTTCGGAGCAAACAGCATCTAAAGAGCTGCGACCCAACT
GCAGCCTAAAAAAATCAAACCTGCTCATGCACCATGGTTGTTCAAACTCGGTTCCCTTCGTGGATTATTTT
GTGTTACATCTGGCTGCTTGGCTTTGCACACACGGGGGAGGCGCAGGCTGCGAAGGAAGTACTATTACT
GGACTCGAAAGCACAACAAACAGAATTGGAATGGATTTCCTCTCCACCCAGTGGGTGGGAAGAAATTAGT
GGTTTGGATGAGAACTACACTCCGATAAGAACATACCAGGTGTGCCAGGTCATGGAGCCCAACCAGAAC
AACTGGCTGCGGACTAACTGGATTTCTAAAGGCAACGCACAAAGGATTTTTGTAGAATTGAAATTCACCTT
GAGGGATTGTAATAGTCTTCCCGGAGTCCTGGGAACTTGCAAGGAAACGTTTAATTTGTACTATTATGAAA
CAGACTACGACACCGGCAGGAATATACGAGAAAACCTTTATGTTAAAATAGACACCATTGCTGCAGATGA
AAGTTTCACACAAGGTGACCTTGGTGAAAGAAAGATGAAGCTGAACACTGAGGTGAGAGAGATTGGACCT
TTGTCCAAAAAGGGATTCTATCTTGCCTTTCAGGATGTAGGGGCTTGCATAGCATTGGTTTCTGTCAAAGT
GTACTACAAGAAGTGCTGGTCCATTGTTGAGAACTTAGCTGTCTTTCCAGATACAGTGACTGGTTCGGAAT
TTTCCTCCTTAGTCGAGGTCCGTGGGACATGTGTCAGCAGTGCCGAGGAAGAGGCAGAAAATTCCCCCA
GAATGCATTGCAGTGCAGAAGGAGAGTGGCTAGTACCCATTGGAAAATGCATCTGCAAAGCAGGCTATCA
GCAAAAAGGGGACACTTGCGAACCCTGTGGCCGCAGGTTCTACAAATCTTCCTCTCAGGATCTCCAGTGT
TCTCGTTGTCCAACCCACAGCTTCTCTGACCGAGAAGGATCATCCAGGTGTGAATGTGAAGATGGGTACT
ACAGAGCTCCTTCTGATCCACCATACGTTGCATGCACGAGGCCTCCCTCTGCACCACAGAACCTTATTTT
CAATATCAATCAAACGACTGTAAGTTTGGAATGGAGTCCTCCGGCTGACAACGGGGGAAGAAACGATGTC
ACCTACAGAATACTGTGTAAGCGGTGCAGTTGGGAACAGGGAGAATGTGTGCCATGCGGAAGTAACATT
GGATACATGCCCCAGCAGACGGGATTAGAGGATAACTATGTCACTGTCATGGACCTACTTGCCCATGCAA
ATTACACTTTCGAAGTTGAAGCTGTAAATGGAGTTTCGGACTTAAGCAGATCCCAGAGGCTCTTCGCTGC
TGTTAGCATCACCACCGGTCAAGCAGCTCCCTCGCAAGTGAGTGGAGTCATGAAGGAGCGAGTACTGCA
GCGGAGTGTGCAGCTTTCCTGGCAGGAGCCGGAGCATCCCAATGGAGTCATCACGGAATATGAAATCAA
GTATTATGAGAAAGATCAACGGGAAAGGACGTACTCAACACTCAAAACCAAGTCCACCTCCGCCTCCATT
AATAATCTGAAACCGGGAACAGTGTACGTCTTTCAGATCCGGGCGGTCACTGCTGCCGGTTATGGAAACT
ACAGCCCTAGGCTTGATGTTGCCACACTTGAGGAAGCTTCAGGTAAAATGTTTGAAGCGACAGCAGTCTC
CAGTGAACAGAATCCTGTCATCATAATTGCTGTAGTGGCTGTAGCAGGGACCATCATCTTGGTGTTCATG
GTGTT
CGGCTTCATCATTGGAAGAAGGCACTGTGGTTATAGCAAGGCTGACCAAGAAGGGGATGAAGAACTCTA
CTTTCATTCTTTAGTAACAAATGAGCACCTGTCAGTTTTATAAACCGCAACAATAACTGTTTAAGACAATCA
ATTTTGGATAAACAATCAACTACAGCAGAATAAATCAAGATTTTTAAGTCCCATTTTCCTTTATACATTCTG
CTTATTTTGTTGTTATATGTTTATTTTTTAAACTCTGATCTTGATTGAATGTGATACCATAAGCACAGTTAGG
CTGCAGTGTAAATATATAAAGACATTGTTCTGAGAGCAGTACGATTTCATGGAAAGATTGTTTGGTGGCTT
TGTTAAAATTAATAAAGAATTTTTAAGGATATAGTGTAATTTTCTTCATTGCATTAATATAACCAAATATGCC
TACCTATCTTTGTCTTGAACCAAATGAATAGATTTGGAATACTTTATTGTAATTGAATTTGATATAAAGTTGA
CTGAGCATTTATGTGTTACCTGCATGCTTCTGGGTGCATTGAAATATTTTAACTTTTAAAATGATACTATGT
TGTTTCAATTTTGACTACCTTTTGTGAGGCATACTGGCTACCTCCTCCTATTAGCTAAGATCTTCCAAAGCC
TTATAATGAAAAGTTTATATAAACCATTTCTCTTTCAAATCACTGTCATACTTGGTCACGGATCCCAGGAAT
ATTGTAAATTTTCTAATTTACTCTGCACTTTGTATATCCAGCCTCTATTACCCTCAAGGTGAATATAAAACTA
TGTCTTTTGAATATTTCTCTTTGATTTTGTGATAGCAGTCCCTCATATCTTGTACTAATTTTATGTATATGTC
AACAGTGGTTGGTCTTTAAAAATAAATCAAAGAATAAGTAAGATGTCTAAATGTTTTTAATATTGCTGTCAG
TGTAGTGGTCTACCTGTAAATGAAATGTCTTATTTCCCTGGTGTATTAATTTGATGGATTGACTGGCTGATA
GATTTATCAATGTGTCTACAAGTCCATTCCAACCCTCGGTATGGCATGAAGTAAGCTGTGATGGCTCTGA
GTTTAATCAAAACCTCCGGTCCGATAGGCACAGGCACGTCAGAAATTTCGGTGGATGTCTTTTGTTGATTT
ACTGGAAAAGCAATTCAGTTAGTAACCTATGAGTTGGATC
CTATGCTACCTATAGAAGCTTGCACATAATACGTAGACGCCAGCAGACTGGAGTAGTCTGTTAAAACTGC
TGTGAATGAGGTGATGTGGAAGCAAGGTCACTGTTCCAAGGAGCACTCTGCA

EphrinA5

TTGATTCGAGATCCATTGTGCTCTAAAGATTTTCCGCATCCGTCTCCCCCGATGCCGCAGTG
GAGATGCTGCTGCTGGCGGTCGCGGCGCTGTGGGTGTGCGTCCGCGGGCAGGAGCCCGCC

GCAAGCCGGTGGCCGACCGCTACGCCGTCTACTGGAACACCACCAACCCCAGATTCCAGCA
GGGAGACTACCACATCGATGTGTGCATCAACGACTACCTGGATGTATTCTGCCCTCACTATG
AAGACTCGGTGCCAGAAGATAAGACCGAACGCTATGTTCTGTACATGGTGAACTTTGATGGC
TACAGTCCTGCGATCACATTTCCAAAGGGTTCAAGAGGTGGGAGTGCAATCGGCCACACTCC
CCGAATGGACCGCTGAAGTTCTCAGAAAAATTCCAGCTCTTCACACCCTTCTCACTAGGATTT
GAGTTCAGGCCAGGCCGGGAGTATTTCTACATCTCTTCTGCGATCCCAGATAATGGAAGGAG
ATCCTGTCTAAAGCTTAAGGTCTTTGTGCGACCAGCAAACAGCTGTATGAAAACTATAGGTGT
TCATGATCGTGTTTTCGATGTTAACGACAAAGTAGAAAATTCATTAGAACCAGCAGATGATAC
CGTGCGTGAGTCAGCCCGAGCCATCCCGTAGGTGAGAACGCGGCACAGACACCGAGGATA
CCCATCCGGCTTTTGGCAACTCTATTGTTCCTCCTGGCAATGCTTTTGATATTATAGCACAGT
ATGCTCCAGCAACCTGTCGAAGAAAATTTCAGGGTCTTGGAACATCAAAGATCCACCTAACT
GCTCATCCCAAGAAAGGGACCTTTAGAGCACAATGGATCGCGAATCGAATTCCTGCAGCCC
